# A low-cost modular PCB assembly for functional and in-circuit testing

**DOI:** 10.1016/j.ohx.2026.e00844

**Published:** 2026-09-13

**Authors:** Geu M. Puentes-Conde, Ernesto Sifuentes, Javier Molina, Francisco Enríquez-Aguilera, Gabriel Bravo, Alejandra Holguín Ávila

**Affiliations:** Institute of Engineering and Technology, Universidad Autónoma de Ciudad Juárez (UACJ), Ciudad Juárez 32310, Mexico

**Keywords:** In-circuit testing (ICT), Functional testing (FCT), Printed circuit board assembly (PCBA), Device under test (DUT)

## Abstract

This paper presents the design and implementation of a modular, low-cost printed circuit board (PCB) assembly to perform in-circuit testing (ICT) and functional testing (FCT) of electronic assemblies during prototyping and small-scale production. The hardware integrates multi-parameter measurement capabilities—including multiple testing functions, programmable voltage stimuli, relay-based switching matrices, voltage/current/resistance/capacitance measurements, and functional signal testing, all coordinated by an embedded MCU on a modular PCB assembly.

Unlike conventional modular ICT/FCT platforms, the system emphasizes architectural integration, reduced structural complexity, and ease of deployment in resource-constrained environments, including small- and medium-sized enterprises (SMEs) and in-house prototyping laboratories. The design rationale, hardware architecture, and implementation are described in detail, along with representative experimental validations that demonstrate functional and practical measurement ranges.

All design files, including schematics, PCB layouts, firmware, and a bill of materials, are openly available to support reproducibility and reuse. Prototypes were built and tested in automated functional test setups to verify their performance and proper operation. The results show that a modular, low-cost PCB assembly can effectively carry out testing tasks typically handled by expensive commercial systems, providing a versatile and low-overhead alternative to end-of-line test equipment.

## Graphical abstract

**Figure:**
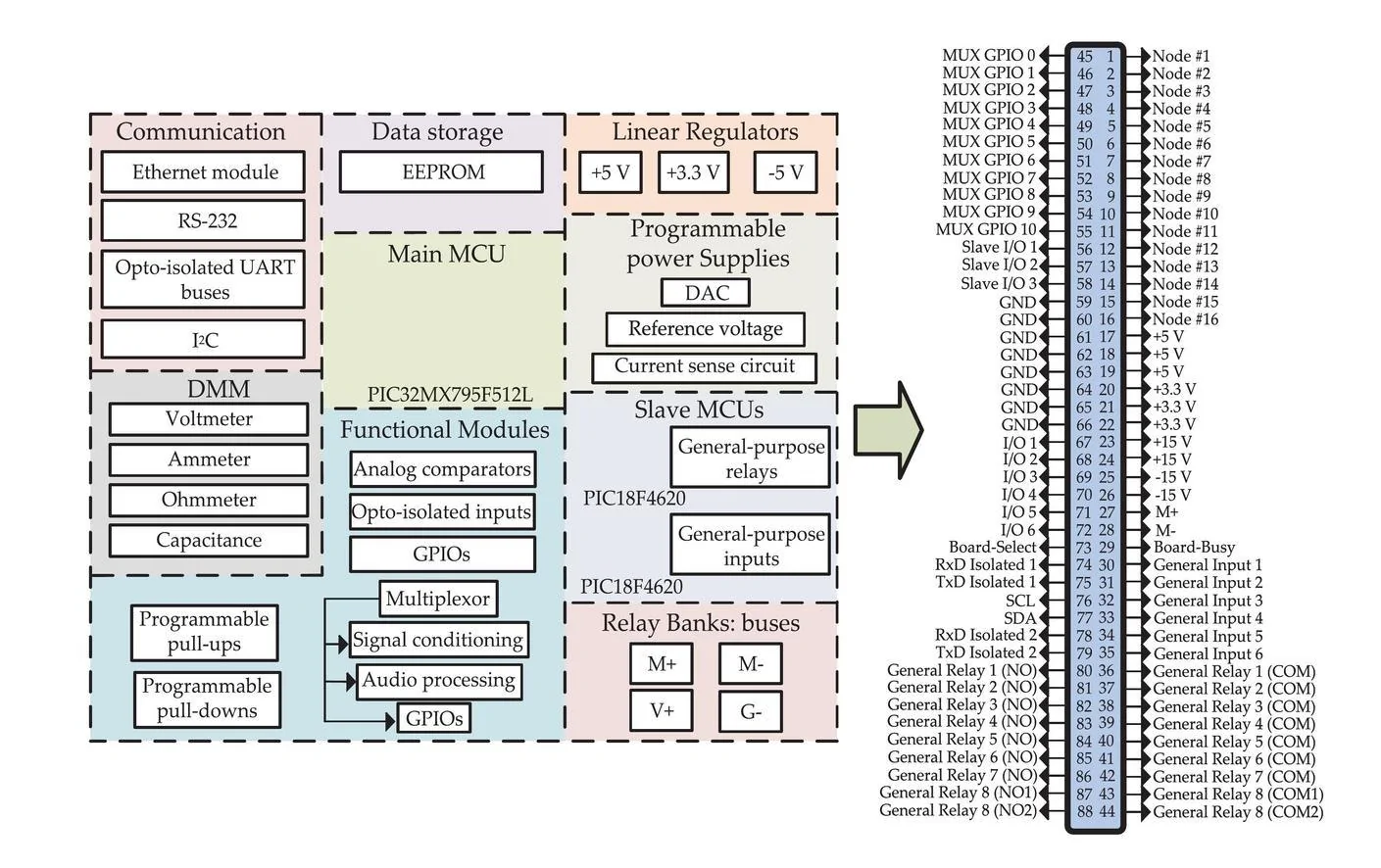

## 1 Specifications table

**Table:** 

Hardware name	Modular PCB assembly: Electrical Testing Board
Subject area	• Engineering and materials science • Educational tools and open-source alternatives to existing infrastructure
Hardware type	• Electrical engineering and computer science
Closest commercial analog	• Commercial in-circuit systems, such as Keysight Technologies, and modular in-circuit testing systems.
Open source license	• Hardware: CERN Open Hardware License (OHL) v2.0 – Permissive • Software: GNU General Public License (GPL) v3.0 • Documentation: Creative Commons Attribution 4.0 International (CC-BY-4.0)
Cost of hardware	∼ $790 USD
Source file repository	https://doi.org/10.17632/2vr3p4nh7d.2

## 2 Hardware in context

This paper presents a detailed description of a modular, cost-effective printed circuit board assembly designed to perform in-circuit testing (ICT)—which verifies individual electronic component values and structural integrity—and functional testing (FCT), which validates the overall operational behavior of the system, both methodologies evaluate the integrity of electronic components and circuitry of an electronic product, commonly referred as Device Under Test (DUT) [1]. Industry adopts these diagnostic methodologies to address defects in the manufacturing process of electronic products. Defects can arise from various factors, such as improper material handling, component quality issues (e.g., errors in parametric tolerances or internal defects), assembly failures in SMT equipment, or human error [2]. These methodologies are indispensable tools that ensure manufactured products meet the required electrical specifications and tolerances [3], [4]. Some industrial-grade commercial systems developed by leading manufacturers such as Keysight Technologies, Agilent Technologies, Circuit Check, Teradyne, and SPEA, among others, perform electrical testing, measuring parameters such as voltage, current, resistance, capacitance, inductance, and short- and open-circuit detection. In summary, these systems convert analog signals to a digital form. In an analog in-circuit test, the system undergoes various conditioning stages in which a voltage (*V*) or current (*I*) stimulus is applied via testing nodes, and the resulting signal is routed through multiplexers and subjected to signal conditioning, including amplification, filtering, and linearization. Finally, an analog signal is digitized as an electrical parameter [1].

The analysis of these highly specialized systems, which offer high precision and reliability in fault detection, reveals a lack of compact, low-cost alternatives suitable for small- and medium-sized industries, as their cost typically ranges from $ 25 k to $ 250 k USD due to system complexity. Table 1 shows the common architecture of a highly modular In-Circuit test system with multiple modular hardware integrations, such as primary modules, cards, and subsystems whose core functions can be broken down. To circumvent the high capital cost and structural overhead typical of these industrial platforms, recent literature has explored alternative testing paradigms. Recently, Lai et al. [5] developed a cost-effective machine learning model to accurately predict resistance, inductance, and capacitance, thereby verifying the integrity of analog IC packages without sole reliance on time-consuming physical testing. Similarly, targeting development barriers, Monagas et al. [6] proposed a modular in-circuit testing system tailored for cost-effective prototype evaluation. While valuable, these contributions fundamentally align with traditional commercial paradigms by focusing primarily on software modeling and modular hardware integration.

**Table 1:** Hardware architecture of commercial ICT/FCT systems [7].

Module	Card	Capabilities
System control	Control Card	Coordinates communication between the master host controller and the machine.
	Analog Stimulus Response Unit	The core metrological instrumentation of the system, programmable analog stimulus sources, and synchronous detectors enable the quantitative evaluation of resistors, capacitors, inductors, and analog integrated circuits.
Testhead interface	Multiplexed Cards	A multiplexed architecture leverages a high-bandwidth switching matrix to map localized instrumentation resources to multiple destination nodes dynamically.
	Analog-Only Pin Cards	A card used for measuring passive components such as resistors, capacitors, and inductors.
	Hybrid Double-Density Cards	They handle digital testing and analog testing on the same pins.
	Digital-Only / High-Speed Digital Cards	They handle high-speed logic, boundary scan (JTAG), and complex digital IC testing.
Power control	System Power Supplies	These programmable, high-reliability DC power cards provide the primary voltage rails to the target DUT. Characterized by high-precision telemetry, they feature hardware-implemented over-voltage (OVP) and over-current (OCP) mitigation frameworks to protect the system.
	Power Control Card	This card orchestrates the deterministic sequencing, controlled voltage ramping, and low-latency emergency shutdown of both the system and DUT power distribution networks.
Auxiliaries	−	Boundary scan testing and vectorless test modules by measuring microfarad-level capacitance between an external sensor plate placed over an IC and the pins beneath it.

To address these limitations, this paper introduces a compact, cost-effective Electrical Testing Board tailored for small- and medium-sized enterprises. Serving as a versatile instrumentation platform throughout the electronic product development cycle, this modular architecture facilitates seamless integration into automated test equipment environments while incorporating fixture test analysis methodologies [8]. Notably, the proposed hardware is not intended as a direct substitute for a high-volume, industrial-grade ICT/FCT systems. Rather, it is positioned as a low-cost, integrated, and reproducible open-hardware alternative designed to support circuit prototyping, academic instruction, and small-scale manufacturing where full-scale ICT/FCT infrastructure remains economically unfeasible.

## 3 Hardware description

**Fig. 1:**
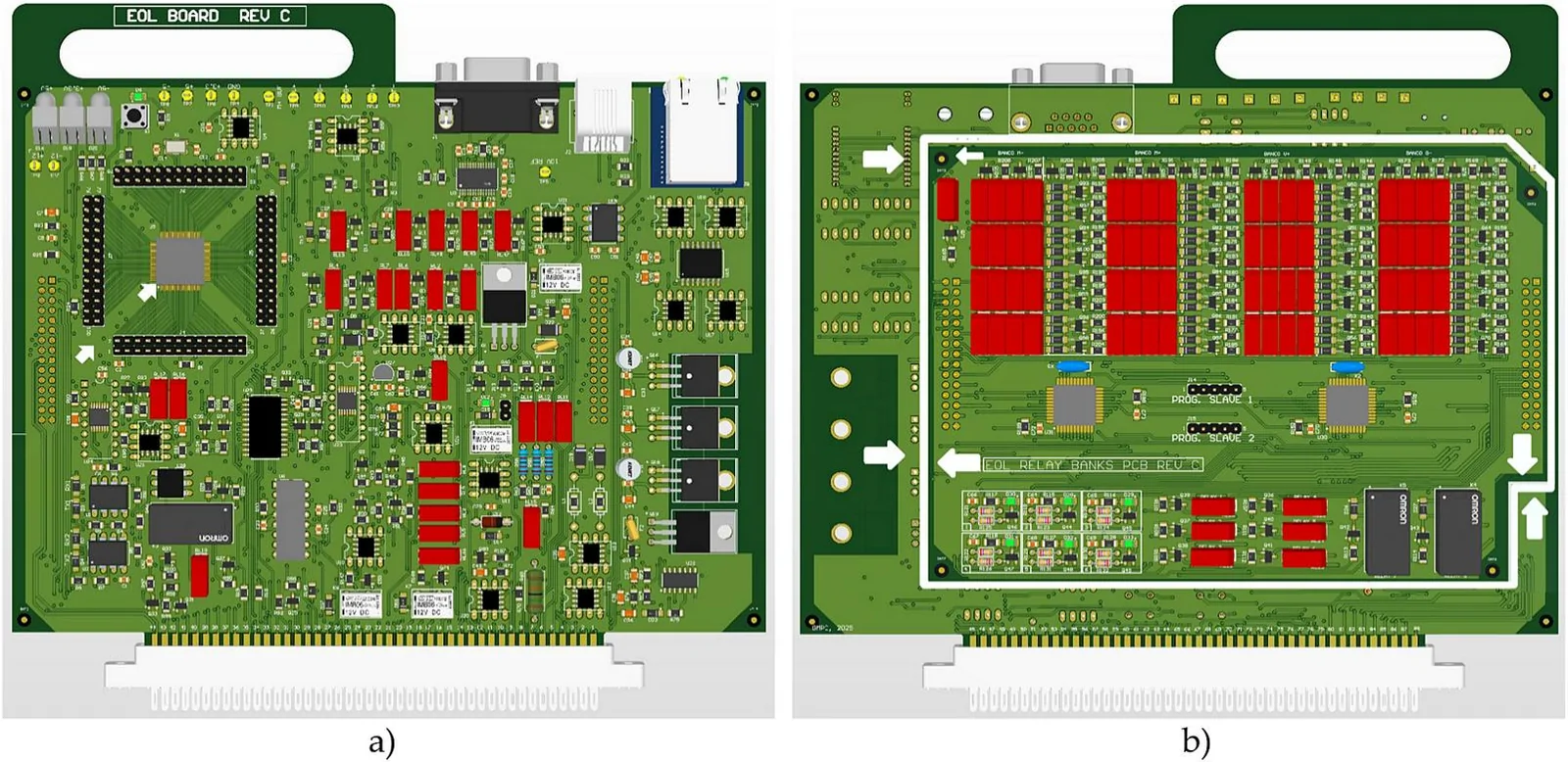
Low-cost electrical testing board. (a) Top side and (b) bottom side.

The modular PCB assembly, named the Electrical Testing Board, is shown in Fig. 1. It is fabricated on a four-layer FR-4 substrate with a total thickness of 1.6 mm and overall dimensions of 187 x 160 mm, featuring an integrated handle at the top and a gold-finger edge connector at the bottom for backplane integration. This assembly interconnects the test probe nodes of a DUT via the edge connector to perform automated in-circuit analog testing (ICT), including resistance and capacitance measurements and open- and short-circuit detection. Furthermore, the PCB assembly supports functional testing (FCT) capabilities, including voltage stimuli, digital multimeter (DMM) measurements, analog signal amplification, audio detection, and data transceiver operations.

**Fig. 2:**
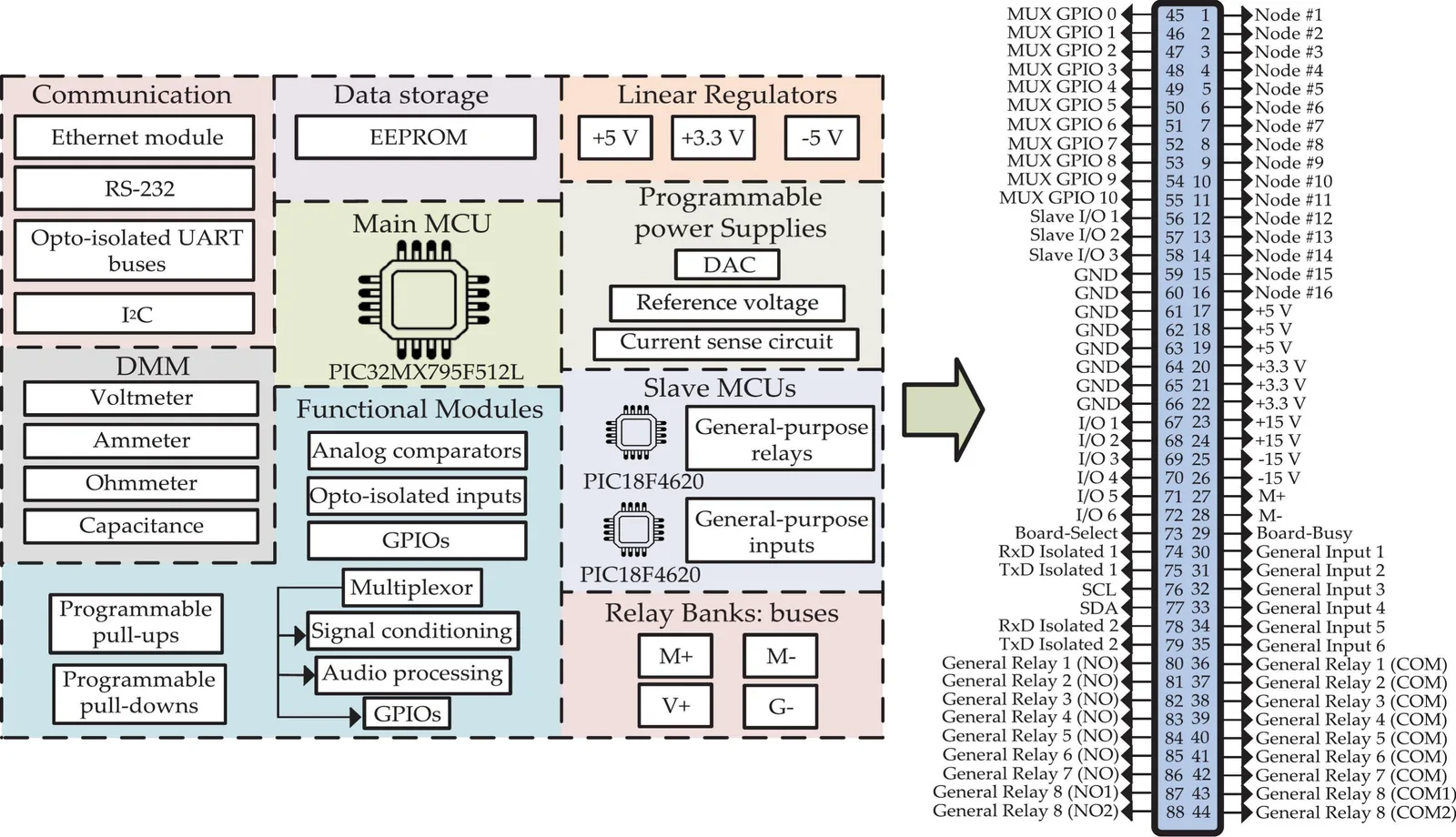
Electrical testing board architecture and its gold-finger edge connector.

Test execution on the Electrical Testing Board relies on direct connectivity between the DUT nodes and the system’s gold-finger edge connector. The overall system architecture is detailed in the block diagram of Fig. 2; each functional block is interconnected via relay banks, also known as relay-based switching matrices.

Fig. 2 provides a detailed breakdown of the 88-pin gold-finger edge connector. The connector links directly to the power management module, which receives main supply inputs (±15 V) to power-up the modular PCB assembly. Onboard low-dropout (LDO) regulators derive ±5 V and +3.3 V rails from these inputs to power the integrated circuits, signal-conditioning networks, and analog stimulus circuits. Connectivity to the DUT is achieved via sixteen available test nodes. These nodes can be mapped or tied concurrently to any of the four signal buses to interact with the ICT/FCT modules. This bus assignment is handled by four relay-based switching matrices (M+, M−, V+, and G− buses) under the control of two slave MCUs, providing complete signal routing across the Electrical Testing Board.

The edge connector also routes two opto-isolated UART buses, an I^2^C bus, eleven multiplexed GPIO lines ('Address') to the M+ bus, and two direct finger contacts to the M+ and M− buses.

Furthermore, a pluggable PCB houses the main MCU—a Microchip PIC32MX795F512 operating at 80 MHz—which controls the test modules via GPIO, I^2^C, and SPI interfaces to initiate testing. To ensure rapid field serviceability, the core module can be replaced in seconds without desoldering tools if the MCU fails. It also provides six optional digital I/Os, plus two dedicated lines for Electrical Testing Board control: a digital input for board selection and a digital output for board-busy status. The main MCU controls two slave PIC18F4620 MCUs via I^2^C. These MCUs control the relay-based switching matrices, monitor six general high-voltage inputs for low-voltage transitions, drive eight general-purpose relay outputs with high-current capabilities, and provide three optional general-purpose digital inputs/outputs.

Table 2 summarizes the operational capabilities of the Electrical Testing Board and the testing methodologies it supports, using the block modules shown in Fig. 2 as a reference framework. Subsequent sections present a comprehensive analysis of the electrical circuit design associated with each gold-finger interface.

**Table 2:** Electrical testing board capabilities and test coverage.

Block module	Modular PCB assembly capabilities
Opto-isolated UART and RS-232	Safe data transceiver operations between DUT and the Electrical Testing Board.
	Data transceiver operations between the host controller (e.g., a computer or PLC) and the Testing Board.
I^2^C bus	Data transceiver operations between DUT and the Electrical Testing Board.
Ethernet	TCP/IP data transmission between a host controller (e.g., PC, PLC) and the modular PCB assembly.
Testing nodes	Sixteen testing nodes can interconnect the DUT nodes to the relay-based switching matrix for testing.
Relay-based switching matrices	A V+ bus to connect high-current voltage stimuli to test nodes.
	M+ and M− buses to interconnect multiple functional blocks to perform a range of testing tasks.
	A G- bus to interconnect test nodes to ground, guard circuitry, and set up the ammeter circuitry.
Programmable power supplies	A high-current power supply (VDUT) from 0 V to 10 V, with pre-regulator and overcurrent protection circuitry, for general voltage stimuli to the DUT via the V + bus.
	A high-current power supply (DMM_Supply) from 0 V to 10 V, with pre-regulator circuitry, that can be interconnected to the M+ bus for general voltage stimuli.
	A reference analog voltage (CROSS_REF_VOLT) can be set as the reference voltage for the functional block on the M+ and M− buses.
	An analog voltage (CURRENT_PROG) to set an analog current limit in the overcurrent protection circuitry for the VDUT power supply.
Analog comparators	Short-circuit and open-circuit detection.
Pull-ups and Pull-downs	Programmable pull-ups and pull-downs to tie a high or low logical voltage for testing purposes.
DMM	Voltmeter measurements range from 0 to 10 V and from 0 to 100 V.
	Ammeter measurements range from 1 µA to 1 mA and can be set by user-defined ranges.
	The ohmmeter measurement range is 0.5 Ω to 1 MΩ.
	Capacitance measurement ranges from 10 nF to 220 µF.
Signal amplification	Programmable analog signal amplification.
Audio conditioning	Analog voice processing for voice pattern recognition and horn frequency patterns.
General-purpose inputs/outputs	General relay outputs with high-current capability, ranging from 500 mA to 2 A across relay contacts.
	High-voltage general-purpose inputs to a low-voltage transition.
Main MCU GPIOs	Optional digital general-purpose input/outputs.
	A digital input to indicate board selection for testing purposes.
	A digital output to indicate the board assembly is busy for testing purposes.
Slave MCU GPIOs	Optional digital general-purpose input/outputs.
Multiplexor	Eleven multiplexed GPIOs to the M+ bus.
Opto-isolated inputs	Analog voltage inputs through an opto-isolated circuit on the M+ and M− buses to detect voltage transitions without direct wiring to the device board assembly circuitry.
Speed-up circuit	Circuitry capable of generating a Pulse Width Modulation (PWM) output signal on the M− bus with an adjustable duty cycle for testing purposes.
EEPROM	A 2 K-bit Electrically Erasable Programmable Read-Only Memory (EEPROM) able to store parametric data and relevant testing information via I^2^C with the main MCU.

To establish the operational boundary conditions of the proposed architecture relative to its metrological capabilities and test coverage, Table 3 summarizes the practical performance parameters, timing constraints, and physical limitations of the modular PCB assembly, thereby delineating its application scope for small- and medium-sized enterprises (SMEs) and in-house prototyping laboratories. In this context, the modular PCB assembly accessibility and operational deployment are strictly defined in terms of open-hardware transparency, economic feasibility, and architectural integration by domain experts in electrical engineering, test automation, and academic research, rather than turnkey usability by non-specialized personnel, which is specified by the target sector.

**Table 3:** General electrical testing board limitations.

Category	Considerations
Scalability & Topology	Sixteen available test nodes per modular PCB assembly.
	Single-DUT execution (no parallel DUT testing per module); sequential ICT/FCT is enforced via the relay-based multiplexing switching matrices.
	Scalable up to 12 daisy-chained boards per host controller, requiring a dedicated digital output signal per module for board selection addressing.
Timing & Throughput	Relay configuration and settling time: The Coto 9012–12-11 reed relay exhibits an electromechanical operating time of 0.35 ms (typical), including a contact bounce duration of 0.1 ms. To ensure dynamic contact resistance stabilization and eliminate switching transients, a firmware-enforced settling delay of 1.0–2.0 ms is required before initiating ADC acquisition.
	Maximum ADC throughput: 100 kSPS (10 µs conversion cycle time, 16-bit resolution).
	Typical ICT/FCT measurement cycle time per DUT is around 10 ms–50 ms (encompassing relay matrix configuration, settling time, signal excitation, and ADC sampling).
Signal Integrity & Hardware	The raw analog front-end bandwidth is 1 MHz at full power, with a maximum Nyquist frequency of 50 kHz at 100 kSPS. Still, the practical, useful analog bandwidth of the modular PCB assembly is deliberately limited to DC–10 kHz (−3 dB) due to the low-pass *RC* network formed by relay matrices in the ON state (*R*_ON_).
	Systematic offset error in low-resistance measurements (*R* < 10 Ω) induced by relay channel on-state resistance (*R*_ON_ = 150 mΩ typ., ≤ 250 mΩ max.). Mitigated via firmware zero-calibration (tare subtraction).
Coverage & Constraints	Bounds the onboard programmable excitation source for ICT/FCT rail supply (0.0 V to 10.0 V_DC_). Limits functional testing to low-voltage electronics (1.8 V, 3.3 V, 5.0 V, and 9.0 V) logic/analog rails.
	To manage multi-board daisy-chained architectures from a host controller, a commercial multifunction data-acquisition interface is required to orchestrate digital handshaking, monitor hardware busy flags, and govern execution sequence selection.
	Inductance measurement is currently unsupported because the onboard signal generator lacks AC impedance excitation and phase-detection circuits.
	Unsupported IEEE 1149.1 (JTAG) boundary-scan capabilities.
Target Sector	Modular PCB assembly is not certified for industrial deployment, limiting its operational scope strictly to controlled laboratory, prototyping, and small-scale production environments for engineering personnel experts.

Additionally, to contextualize the technical viability and operational boundaries of the proposed hardware, Table 4 presents a quantitative comparative synthesis against the modular ICT platform reported by Monagas et al. [6] and an industrial-grade commercial ICT system (Keysight i3070 Series) [7]. The developed system is benchmarked head-to-head against key performance indicators—spanning signal excitation ranges, measurement ranges, switching latencies, structural digital coverage, and economic metrics—and this comparative analysis establishes the trade-offs between low cost and modular scalability.

**Table 4:** Architectural and economic comparison of proposed hardware with other systems.

Feature	Proposed modular PCB assembly	Monagas et al. (modular ICT platform)	Industrial-grade commercial system (Keysight i3070 Series)
Approximate base cost	∼$790.00 USD (fabrication of the proposed hardware)	∼3000.00 EUR (complete base cost system)	$25 k–$250 k USD (complete base cost system: instrument cards, chassis/rack, enclosure, etc.)
	∼$3500.00 USD (estimated complete system cost: proposed hardware, host controller, enclosure, cables, test probes, etc.)		
System architecture	Modular PCB assembly	Modular: instrument cards	Proprietary large-scale enclosure
			Modular: multiple instrument cards
Footprint / Portability	Low (compact, modular embedded instrumentation)	Chassis enclosure and backplane for modular cards	Stationary racks/chassis enclosure
Licensing / Software	Open-source ecosystem	not reported	Proprietary environment/driver licensing
Target Sector	Small- & medium-sized enterprises (SMEs) and in-house prototyping laboratories.	Small- & medium-sized enterprises (SMEs).	Large electronic manufacturing services (EMS)
Test Nodes	16 per PCB assembly	Expansion modules up to 16, 32, 48 and 64	2-Module system: up to 2,592
Switching Speed: Relay Settling Dynamics	0.5 ms to 2.0 ms actuation time	not reported	≤ 0.5 ms
Data Acquisition Rate & Typical Test-Cycle Time	100 kSPS	not reported	1 MSPS to 10 MSPS
	ICT/FCT around 10 ms to 50 ms	not reported	not reported
Voltage Ranges	Stimulus: 0.0 V to 10.0 V_DC_ (*12-bit DAC onboard excitation*)	Stimulus: 0.0 V to 5.0 V_DC_ (*fixed low-voltage digital/analog logic level*)	Stimulus: ±100 mV to ± 100 V_DC_ (*programmable DC sources*)
	Measurement: 0 V to 10.0 V_DC_ (*ADC input range*) and onboard resistive attenuation for 0 V to 100.0 V_DC_	Measurement: 0.0 V to 5.0 V_DC_*(limited by single-supply MCU ADC bounds)*	Measurement: ±100 mV to ± 100 V_DC_*(high-voltage pin-card rating)*
Resistance Ranges	0.5 Ω to 1 MΩ	not reported	0.01 Ω to 100 MΩ
Capacitance Ranges	10 nF to 220 µF	not reported	1.0 pF to 10,000 µF
Ammeter Ranges	Range Scale I: 1 µA to 10 µA	not reported	Low Range: down to 100nA
	Range Scale II: 10 µA to 100 µA		Standard Range: 10 µA to 100 mA
	Range Scale III: 0.1 mA to 1.0 mA		High Range: up to 750 mA
Digital Test capabilities	fully supported	fully supported	fully supported
Audio Conditioning	Analog voice processing for voice pattern recognition and horn frequency pattern.	unsupported	unsupported
Analog Test capabilities	fully supported	fully supported	fully supported
Short- and Open-Circuit Detection	fully supported	not reported	fully supported
Safety & Protection Features	Hardware level: resettable PTC polyfuses onboard, galvanic isolation in UART port and ESD protection onboard	not reported	Industrial Standards Compliant IEC 61010–1
Boundary-scan / JTAG	unsupported	not reported	Fully supported

### 3.1 Communication approach

The modular PCB assembly supports multiple communication protocols for testing control, including RS-232, direct opto-isolated UART buses, and Ethernet. These protocols allow a host controller (e.g., a PC or PLC) to issue commands that initialize testing tasks, transmit tolerances and measurement limits, request test results, or directly control individual testing blocks via software.

#### 3.1.1 Opto-isolated UART buses

The modular PCB assembly features two opto-isolated UART circuits designed to protect the MCU UART ports from bus collisions or electrical malfunctions. This architecture allows a single UART bus on the host controller to control multiple testing boards in parallel via the edge connector, enabling simultaneous parallel communication for multiple DUTs.

#### 3.1.2 Ethernet module

For network-based control, the modular PCB assembly integrates a Microchip LAN8720 single-chip Ethernet Physical Layer Transceiver (PHY). This module enables direct control of the Electrical Testing Board via TCP/IP commands. This approach is ideal for deployments where the board assembly must interface directly with a PLC or an intranet network without requiring a dedicated local computer.

#### 3.1.3 RS-232 interface

**Fig. 3:**
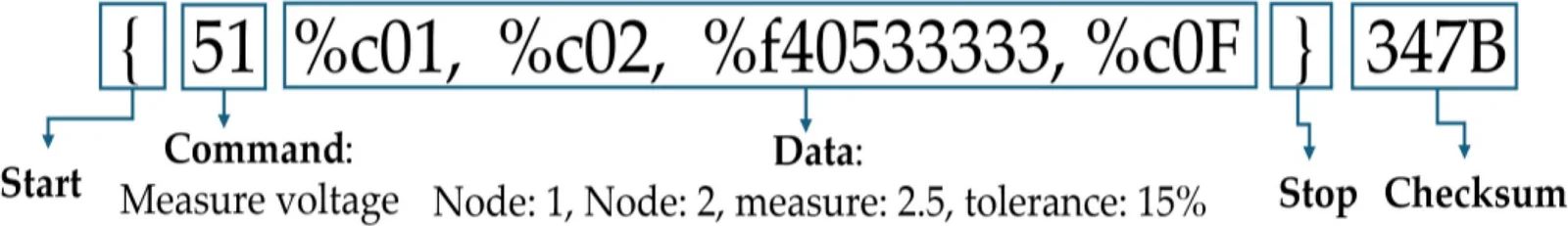
Frame data packet example.

Additionally, a dedicated DB9 connector providing RS-232 communication is included. This secondary interface is primarily utilized for technical diagnostics, debugging, and field maintenance tasks. To establish communication between the Electrical Testing Board and the host controller, a trustworthy communication frame was developed. Commands and testing parameters are transmitted to the Electrical Testing Board for interpretation and execution using the packet structure shown in Fig. 3. The frame is bounded by a 1-byte start delimiter, represented by the open bracket symbol ({), and a 1-byte stop delimiter, represented by the close bracket symbol (}). Within this frame, the command type is defined by a 1-byte field that supports up to 255 unique operational tasks. Fig. 3 shows an example data frame for a single testing task: measuring an analog voltage across selected nodes using arbitrary limit parameters.

Other typical command examples include the following operations:

1. 0x01: Start an automated testing task.
2. 0x02: Send data limits.
3. 0x03: Request data results.
4. 0x50 to 0xAF: Start individual testing tasks, such as short-circuit detection, voltmeter measurement, etc.

Following the command byte, optional parameters may be transmitted as data payloads. The system processes standard data types—such as characters, integers, strings, and floating-point variables—by converting hexadecimal values into their corresponding ASCII representations and floats into IEEE 754-compliant hexadecimal format [9]. When transmitting multiple data items, a comma (,) is used as a delimiter to ensure sequential processing. Table 5 outlines typical data type configurations.

**Table 5:** Data type formatting.

Data type	Identifier	Value	Representation	ASCII conversion
Char	%c	91	hex-format: 0x5B	'5′, 'B'
Integer	%i	13,675	hex-format: 0x356B	'3′, '5′, '6′, 'B'
Float	%f	8.95	float to IEEE754 32-bits: 0x410F3333	'4′, '1′, '0′, 'F','3, '3′, '3′, '3′
String	%s	'b'	no changes	'b'

To ensure data integrity and accurate interpretation, the packet concludes with a 2-byte checksum. This checksum is calculated via a byte-wise summation across the frame. The resulting value is appended to the transmission and compared against the checksum calculated independently by the receiving device to detect transmission errors.

### 3.2 Relay-based switching matrices

Relay-based switching matrices are critical components of the Electrical Testing Board, routing the DUT nodes to multiple integrated testing modules. This routing is managed via four dedicated internal buses, each equipped with its own relay matrix: V+, M+, M−, and G−. This bus-centric architecture yields a compact design footprint while providing the versatility to interconnect any testing module with the required circuitry.

The relay-based switching matrix for the M− bus is configured as an *X*-*Y* matrix and controlled by a slave MCU via GPIO pins. This specific matrix routes sixteen test nodes. The switching matrices for the V+, M+, and G− buses use the same design architecture.

1. V+ Bus: Connects a high-current power supply to the DUT and supports optional discharge operations.
2. M+ and M− Buses: Work in tandem to interconnect multiple functional blocks, enabling a wide range of diagnostic tasks such as voltage stimuli application, digital multimeter (DMM) measurements, analog signal amplification, audio detection, short-circuit detection, and open-circuit testing.
3. G− Bus: Interconnects the DUT nodes to system ground, configures guard circuitry, and establishes the return path for ammeter measurement circuits.

### 3.3 Programmable power supplies

The Electrical Testing Board features a compact, programmable power supply that delivers voltage stimuli to the V+ and M+ buses. The design integrates an Analog Devices MAX536 12-bit voltage-output digital-to-analog converter (DAC) paired with a low-power, low-drift MAX876 10 V precision reference. This configuration establishes a stable reference voltage range of 0 V to 10 V across four independent, programmable internal outputs. Additionally, the architecture incorporates a pre-regulator stage to meet high-current demands and an overcurrent protection circuit that maintains voltage stability by continuously monitoring current.

1. DAQ_1: Generates a high-accuracy, stable analog voltage to configure a high-current power supply (V_DUT_) from 0 V to 10 V via a pre-regulator stage. This channel features hardware-latched overcurrent protection: the main MCU monitors the load current via a digital input, compares it to a programmable threshold, and triggers a hardware shutdown if an overload occurs. This supply routes directly to the V+ bus, serving as the primary voltage source for the DUT.
2. DMM_Supply: A pre-regulated, high-current supply (0 V to 10 V) that connects to the M+ bus to provide general voltage stimuli for automated testing routines.
3. CROSS_REF_VOLT: Generates an adjustable analog reference voltage to drive analog comparators, configurable pull-up networks, and active speed-up circuitry across the M+ and M− buses.

CURRENT_PROG: Establishes a programmable analog reference voltage utilized by the DAQ_1 overcurrent protection circuit to set the hardware current-limit threshold.

Additionally, the V+ bus includes an auxiliary relay circuit to discharge the DUT through a low-resistance, high-wattage resistor. This discharge stage is typically used to protect sensitive measurement circuitry from residual energy stored in capacitive components before subsequent testing phases.

### 3.4 Programmable pull-ups, pull-downs, and input analog comparators

To facilitate dynamic biasing and signal conditioning, the functional module integrated into the M+ and M− buses incorporates software-programmable pull-up and pull-down networks. These networks are instrumented to establish deterministic logic thresholds or reference voltages. The pull-up topology can be dynamically mapped to a selectable potential, driven either by a precision programmable cross-reference voltage supply (CROSS_REF_VOLT) or the regulated 5 V LDO source routed through the M+ bus. Concurrently, the M− bus features dual pull-down channels configurable to distinct, well-defined resistance values referenced to ground. Within the pull-down subsystem, a dedicated Guard connection is implemented via low-insertion-loss relay routing to the G− bus, extending guarding capabilities to any arbitrary node on the DUT. This architecture is critical for isolating low-level current measurements, neutralizing leakage paths, or executing driven-guarding configurations when the target measurement is topologically dependent on adjacent nodes.

In parallel with this network, the M+ and M− buses share a programmable analog comparator module via a relay-multiplexed bus. This subsystem is optimized for high-throughput fault characterization, specifically open- and short-circuit detection. By interacting directly with the programmable power supply, the module establishes a precise analog reference threshold at the comparator inputs. High-speed fault isolation is achieved by injecting a deterministic stimulus voltage into a target node via the V+ bus, while simultaneously acquiring the transient or steady-state response from a coupled node on the M+ and M− buses.

### 3.5 DMM

Multi-parameter electrical evaluation—encompassing voltage, current, resistance, and capacitance—is executed via an integrated DMM subsystem. Dynamic node selection is managed across four dedicated analog buses V+, M+, M−, and G−), thereby facilitating flexible signal routing and measurement isolation.

#### 3.5.1 Voltmeter and ammeter

For voltage acquisition, the primary instrumentation path couples a 16-bit successive-approximation register (SAR) Analog-to-Digital Converter (ADC, AD677) with a low-drift, precision 10 V reference (MAX876). This architecture employs relay-selectable attenuation networks to scale the operational ranges to 0–10 V and 0–100 V. Conversely, an auxiliary, low-latency path uses an internal 10-bit microcontroller ADC to process lower-resolution signals within a constrained 0–3.3 V window when necessary.

Ammeter measurement is executed via a transimpedance amplifier (TIA) topology that maps the input current to a proportional analog voltage, incorporating a forward-biased signal diode (V_forward_) to establish a stable biasing threshold and protect the instrumentation loop. The ammeter subsystem features three discrete gain stages, empirically calibrated to guarantee linear transfer characteristics across the dynamic measurement range. Operationally, the TIA interfaces across the G− bus, modulating the resulting analog signal directly onto the M+ bus for subsequent conversion.

#### 3.5.2 Resistance measurement

**Fig. 4:**
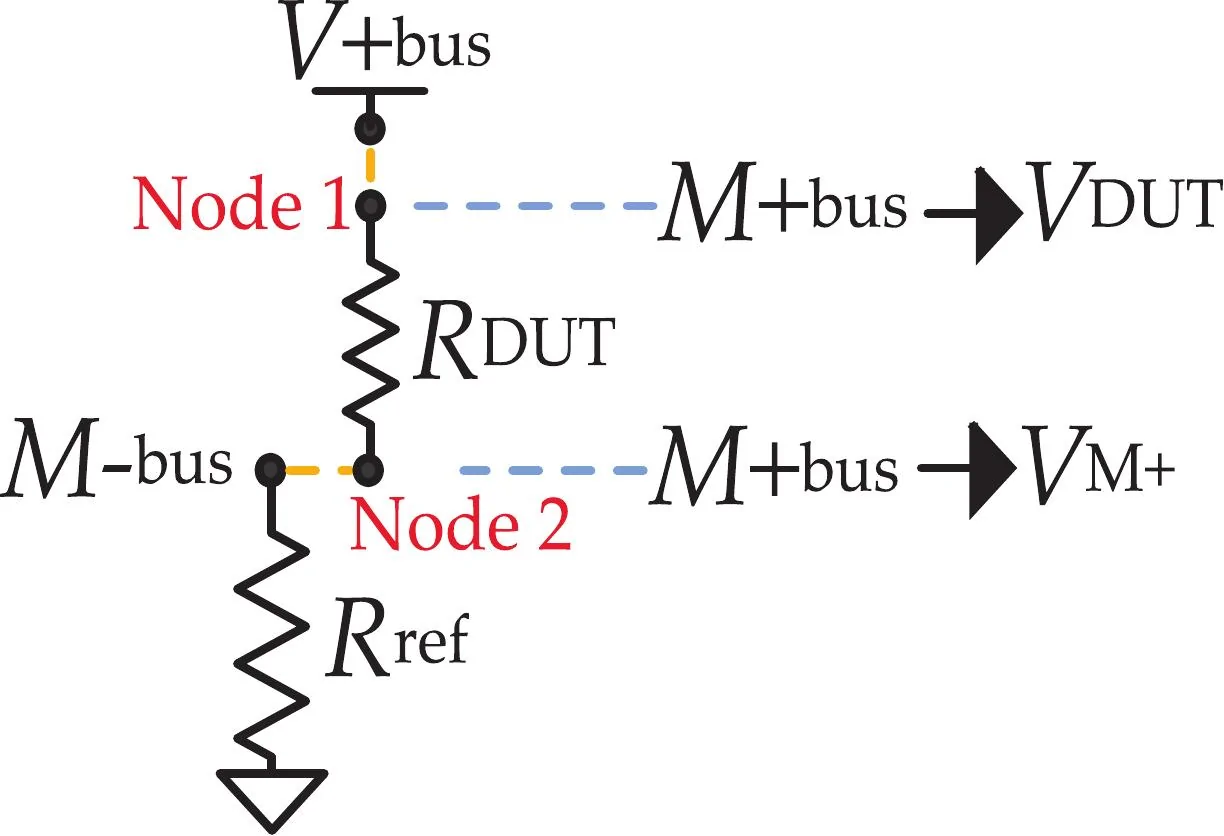
Resistance measurement principle.

The instrumentation architecture further extends to the characterization of resistance and capacitance by systematically coordinating the functional modules across the V+, M+, and M− buses. Resistance estimation relies on a voltage divider network; *R*_DUT_ is set into a ratiometric configuration on board. This is achieved by pairing the R_DUT_ terminals with the test nodes on the V+ and M− buses, which allows the circuit to be completed with the software-programmable pull-downs module, which incorporates low-tolerance reference resistors (*R*_ref_) to bias the DUT’s resistance (*R*_DUT_) and the voltage stimulus subsystem *V*_DUT_, as shown in Fig. 4.

Applying a known stimulus voltage to the network via the V+ bus—denoted within the circuit as *V*_DUT_ —develops a proportional potential across Node 1 and Node 2. The resulting acquisition is subsequently digitized by accessing the respective node paths through the M+ bus. The system’s underlying transfer characteristic is governed by Eq. (1),

(1) $$V_{M+}=V_{DUT}\left(\frac{R_{ref}}{R_{DUT}+R_{ref}}\right)+R_{offset}$$

where *R*_DUT_ is the device under test resistance (Ω), *R*_ref_ is the reference resistance (Ω), *V*_M+_ represents the voltage (V) acquired across the respective node paths via the M+ bus, while *V*_DUT_ denotes the excitation voltage (V) applied through the V+ bus. Rearranging Eq. (1) yields an explicit expression for the unknown resistance *R*_DUT_ as a function of these monitored potentials and the calibrated value of *R*_ref_. To counteract the measurement constraints imposed by the parasitic contact resistance of the relay-based switching matrices in the V+, M+, and M− buses, we introduce an offset resistance, *R*_offset_, to mitigate these confounding effects. The lower bound of the resistance measurement range is physically constrained to 0.5 Ω, dictated by the cumulative parasitic contact resistance of the relay switching matrix. This ratiometric approach offers high computational efficiency and minimizes systematic measurement errors by relying strictly on relative voltage acquisition, thereby ensuring reproducible estimation of the DUT resistance matrix.

#### 3.5.3 Capacitance measurement

The common approach to measure capacitance within an ICT environment, such as the industrial-grade commercial system (Keysight i3070 Series), is to employ In-Circuit Voltammetry paired with Synchronous Phase-Detection. This method operates by routing the target component into an instrumentation loop of a high-precision MOA and applying a programmed AC voltage stimulus to one terminal, forcing a displacement current across the component and into the MOA’s virtual ground, routing it through a known reference resistor to generate an output voltage, then isolate and extract the 90° imaginary component of this output voltage, and effectively filtering out parallel resistive compounding errors and enabling computation of the true capacitance [7].

**Fig. 5:**
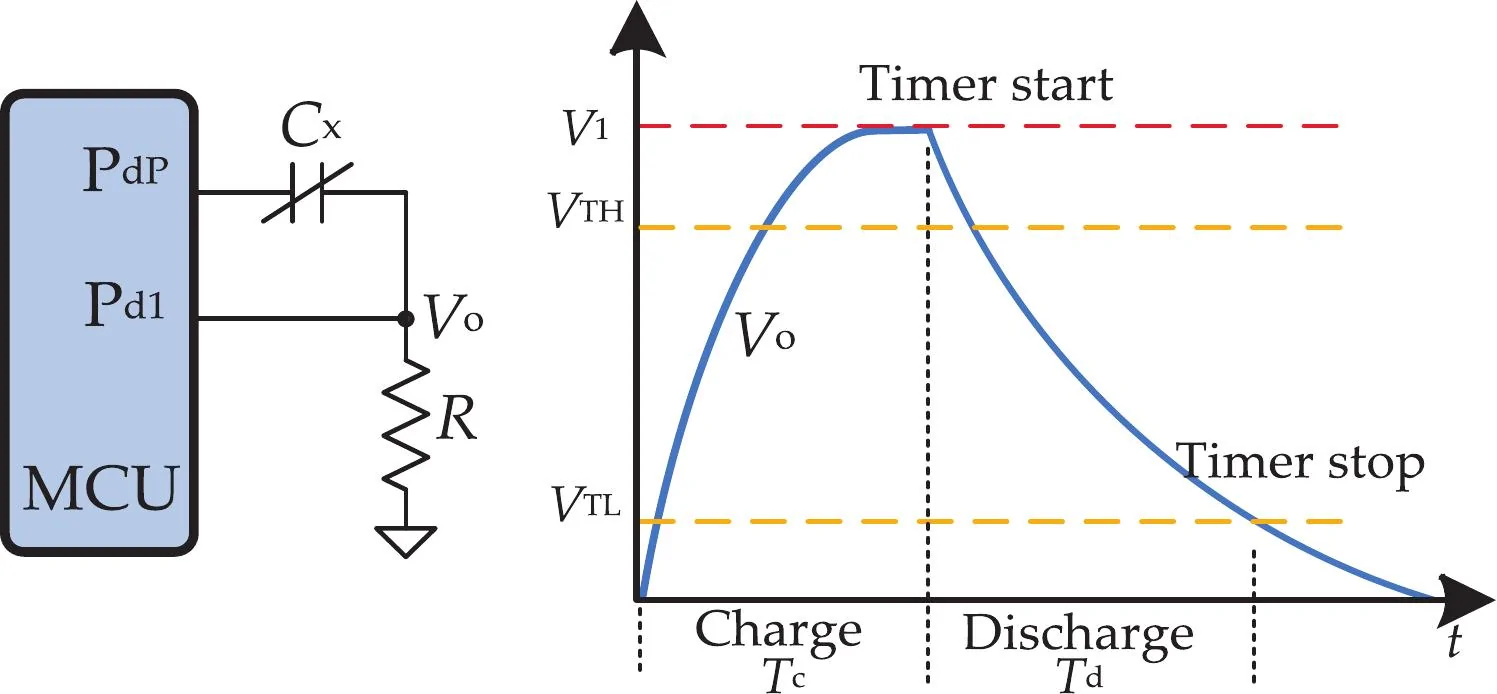
Direct interface circuit for capacitance measurement [10].

To circumvent the structural complexity and high capital expenditure associated with the complex circuitry embedded within commercial-grade ICT systems, contemporary architectures have explored direct sensor–microcontroller interface circuits, commonly designated as Direct Interface Circuits (DICs) [10], [11]. Categorized primarily by discharge-time measurement techniques [12], [13], these topologies leverage discrete *RC* configurations to deduce unknown resistance and capacitance values, as shown in Fig. 5. This framework represents a highly cost-effective measurement paradigm; furthermore, extensive literature validates its efficacy as a viable alternative modality for accurate capacitance estimation. Table 6 summarizes the comparative analysis of the two methods and their features. The operational method in this work relies on measuring the time interval required to discharge (*T*_d_) the capacitor *C*_x_ through the resistive element *R*, until a voltage threshold is reached, *V*_TL_. This time interval serves as the basis for capacitance estimation. The capacitance is directly proportional to the measured time interval. Consequently, the measurement process is performed in two distinct stages: charging and discharging the capacitor *C*_x_.

**Table 6:** Comparative approach for capacitance measurement methods.

Feature	Proposed modular PCB assembly	Industrial-grade system (Keysight i3070 Series)
Primary method	Direct Interface Circuits: *RC* discharge-time measurement	AC voltammetry & phase detection
Core hardware	Standard microcontroller with digital I/O pins	High-precision MOA & synchronous detectors
System cost	Minimal (low-cost embedded systems)	High (capital equipment; multiple instrument cards)
Circuit complexity	Simplified, discrete *RC* network loops	Complex, multi-stage analog instrumentation
Error handling	Mitigates errors via software calibration	Digitally filters parallel resistive errors

This approach provides a highly cost-effective yet reliable method for embedded capacitance estimation within the testing system. The exact digital pin states, transitions, and timing intervals required to complete each stage are detailed in Table 7.

**Table 7:** MCU pin configuration and sequential measurement process for a capacitive sensor [10].

Stage	$P_{d1}$	$P_{dP}$	Process
1	'1′	'0′	$T_{c}=R_{i}C_{x}\cdot ln\left[V_{1}/\left(V_{1}-V_{TL}\right)\right]\approx 5R_{i}C$
2	'HZ', and capture time	'0′	$T_{d}=RC_{x}\cdot \;ln\left(V_{1}/V_{TL}\right)$

*Ri improves noise immunity and is not explicitly shown in Fig. 5.

**Fig. 6:**
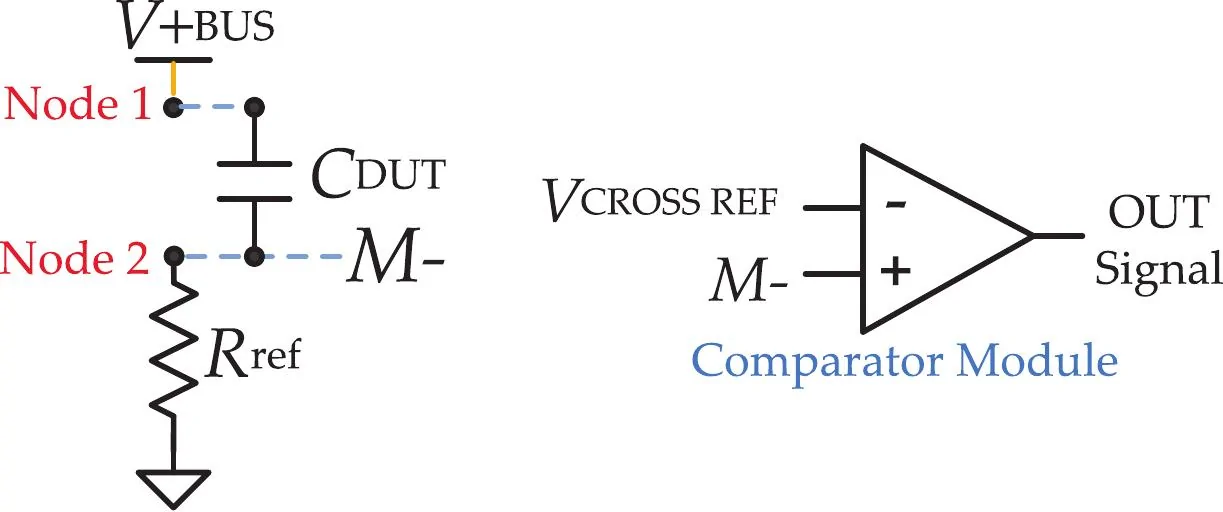
Capacitance measurement principle.

Fig. 6 shows a scheme to estimate capacitance, which is achieved by following the next steps: i) charging the capacitor via the V+ bus in *Node 1* at the voltage stimuli *V*_+bus_ and interconnecting the M− bus to the comparator module, and ii) starting the embedded timer and a discharge-time-based process (*T*_d_) by setting up the pull-down resistor *R*_ref_ in the M− bus. The comparator output stops the embedded timer in the main MCU when the capacitor voltage reaches the cross-reference voltage *V*_CROSS REF_, serving as a comparator threshold to stop the embedded timer and estimate capacitance, solving Eq. (2) in terms of the capacitance *C*_DUT_.

(2) $$T_{d}={(R}_{ref}\cdot C_{DUT})\cdot ln\left(V_{+BUS}/V_{CROSS\;REF}\right)$$

### 3.6 Signal amplification, audio conditioning, and multiplexing

The modular PCB assembly incorporates dedicated signal-conditioning circuitry to process low-amplitude signals. The primary stage features a non-inverting amplifier with a programmable-gain network, dynamically adjusted via an I^2^C-controlled digital potentiometer. Additionally, an audio conditioning subsystem converts acoustic signals into proportional analog voltage levels for amplitude or frequency analysis.

These conditioned signals are routed to the M+ bus through an integrated multiplexer IC for subsequent digitization by the DMM module. This multiplexer also routes eleven general-purpose input/output (GPIO) ports directly to the device board assembly edge connector.

### 3.7 General opto-isolated inputs, speed-up signal, and EEPROM

The modular PCB assembly incorporates an opto-isolated conditioning circuit that interfaces with the M+ and M− buses to detect voltage transitions, providing galvanic isolation and preventing direct electrical coupling to the primary instrumentation. This topology is particularly critical during functional verification phases where the DUT signals must remain structurally isolated from external instrumentation loading to preserve signal integrity.

Additionally, the subsystem incorporates a high-speed driver architecture configured to generate an M− bus Pulse Width Modulation (PWM) excitation signal with a software-adjustable duty cycle for stimulus–response characterization. To facilitate localized, non-volatile data persistence, a 2 K-bit Electrically Erasable Programmable Read-Only Memory (EEPROM) interfaces with the primary MCU via an I^2^C bus, serving as a dedicated repository for hardware calibration vectors, runtime test logs, and device-specific parametric metadata.

### 3.8 General-purpose input/outputs

The modular PCB assembly incorporates an actuation subsystem consisting of eight general-purpose relay outputs with high current-carrying capacity, ranging from 50 mA to 1.5 A across the mechanical relay contacts. Complementing this output stage, the board assembly features a level-shifting input-conditioning topology that converts high-voltage, general-purpose signals into low logic-level voltages compatible with the MCU. This voltage translation step isolates the primary processing unit from transient overvoltages and signal degradation, ensuring robust monitoring of peripheral sensor networks.

### 3.9 Cross-disciplinary applications for laboratory tasks

Beyond the hardware’s primary role for FCT and ICT, the open-hardware architecture provides flexible instrumentation capabilities for researchers both inside (test engineering, electronic manufacturing, hardware prototyping) and outside (bioengineering, materials science, environmental monitoring, academic labs) across various domains with potential uses:

1. Materials scientists and bioengineers can leverage the programmable switching matrices (V+, M+, M−, and G−) to integrate the DMM to execute automated multi-point resistance, capacitance, and impedance mapping across novel conductive polymers, flexible electronics, or biological tissue samples over extended testing periods.
2. Multi-channel precision data acquisition (standard laboratory task): Applied physics and electrical engineering laboratories can utilize the onboard ADCs, programmable attenuation networks (0–10 V and 0–100 V), and wide-range TIA ammeter (1 µA to 1 mA) as a compact, cost-effective substitute for bulky benchtop instruments in automated experimental setups.
3. Isolated sensor node Interfacing for harsh environments (standard/novel): Environmental and industrial IoT researchers can deploy the board as a ruggedized interface layer. The opto-isolated UART channels, opto-isolated analog inputs, and industrial Ethernet (TCP/IP) allow safe monitoring of transducers and high-voltage transitions while preventing ground loops and protecting host controllers.
4. Acoustic and signal-conditioning experimentation (novel task): Research teams working on embedded acoustic processing or biomedical signal acquisition can utilize the onboard programmable signal amplification and specialized audio-conditioning circuitry for real-time voice/frequency pattern characterization and transducer frequency–response evaluation.
5. Custom Experimental Automation and Arbitrary Test Bench Creation: Researchers with an electrical engineering background can leverage the open-hardware architecture—combining programmable power sources (0–10 V), software-configurable pull-up/pull-down networks, 88-pin switching matrices, and general-purpose relay driver outputs—to rapidly configure custom test benches for arbitrary automated tasks, such as stress-testing power electronics, characterization of custom IC prototypes, or dynamic load switching.

## 4 Design files summary

To ensure hardware verification, the Electrical Testing Board was systematically developed within the Altium Designer Professional (v23.1.1) electronic design automation (EDA) environment. The deployment workflow followed a modular implementation strategy: each functional block detailed in Fig. 2 was initially prototyped and validated on an isolated breadboard matrix to verify circuit-topology behavior and signal-integrity boundaries.

Following individual module verification, the complete hardware architecture was integrated into a modular system comprising three distinct PCBs. The comprehensive open-source hardware repository, including schematics, manufacturing files, and firmware assets, is systematically cataloged in Table 8 and hosted within the supplemental compressed file “PCBs and Schematics.zip”.

1. Main PCB: It houses all functional modules to interact with the relay-based switching matrices.
2. Relays PCB: It houses the relay-based switching matrices to interconnect V+, M+, M−, and G− buses to test nodes.
3. MCU PCB. It houses the main MCU PIC32MX795F512 for robustness and ease of serviceability.

**Table 8:** Design source files.

Design file name	File type	Open-source license	Location of the file
PCBs and Schematics	Altium Project	CERN OHL-P 2.0	https://doi.org/10.17632/2vr3p4nh7d.2
PIC32 firmware	MPLAB X Project	GNU General Public License, version 3 (GPLv3)	https://doi.org/10.17632/2vr3p4nh7d.2
PIC18 firmware	MPLAB X Project		https://doi.org/10.17632/2vr3p4nh7d.2
Graphical User Interface	CVI/LabWindows Project		https://doi.org/10.17632/2vr3p4nh7d.2
Hardware BOM	XLSX	CERN OHL-P 2.0	https://doi.org/10.17632/2vr3p4nh7d.2
Debugging enclosure	ZIP files	CC BY 4.0	https://doi.org/10.17632/2vr3p4nh7d.2

The firmware architecture follows a distributed processing topology developed within the Microchip MPLAB X (v6.20) integrated development environment (IDE). For the primary control unit, the PIC32MX795F512L firmware is compiled using the XC32 (v4.60) toolchain. The associated development assets, including editable C source files and definition headers, are compiled within the repository compressed file “PIC32 firmware.zip”.

Concurrently, the firmware for the two auxiliary PIC18F4620 slave MCUs is compiled using the C18 (v3.46) compiler. The corresponding codebase, including all modular source code and header definitions, is in the supplemental compressed file “PIC18 firmware.zip”.

System orchestration and direct control of the Electrical Testing Board are performed via a host PC that serves as the host controller in a master–slave architecture. The dedicated Graphical User Interface (GUI) was developed in ANSI C using the National Instruments LabWindows/CVI integrated development environment. This host interface dispatches deterministic testing routines via RS-232 serial and Ethernet communication to facilitate hardware-level validation, real-time diagnostics, and automated calibration sequences. During calibration, the software computes and transmits localized offset vectors to be committed to the on-board non-volatile EEPROM, utilizing a specialized diagnostic and debugging interface enclosure.

## 5 Bill of materials summary

The Bill of Materials (BOM) for the modular PCB assembly is detailed in the supplementary technical documentation cataloged in Table 8, which specifies component nomenclature, quantities, unit metrics, and individual unit pricing. The aggregate component cost required to build a modular PCB assembly prototype totals $ 790.00 USD, with all components procured through commercial distribution channels (DigiKey).

## 6 Build instructions

### 6.1 Overall system description

**Fig. 7:**
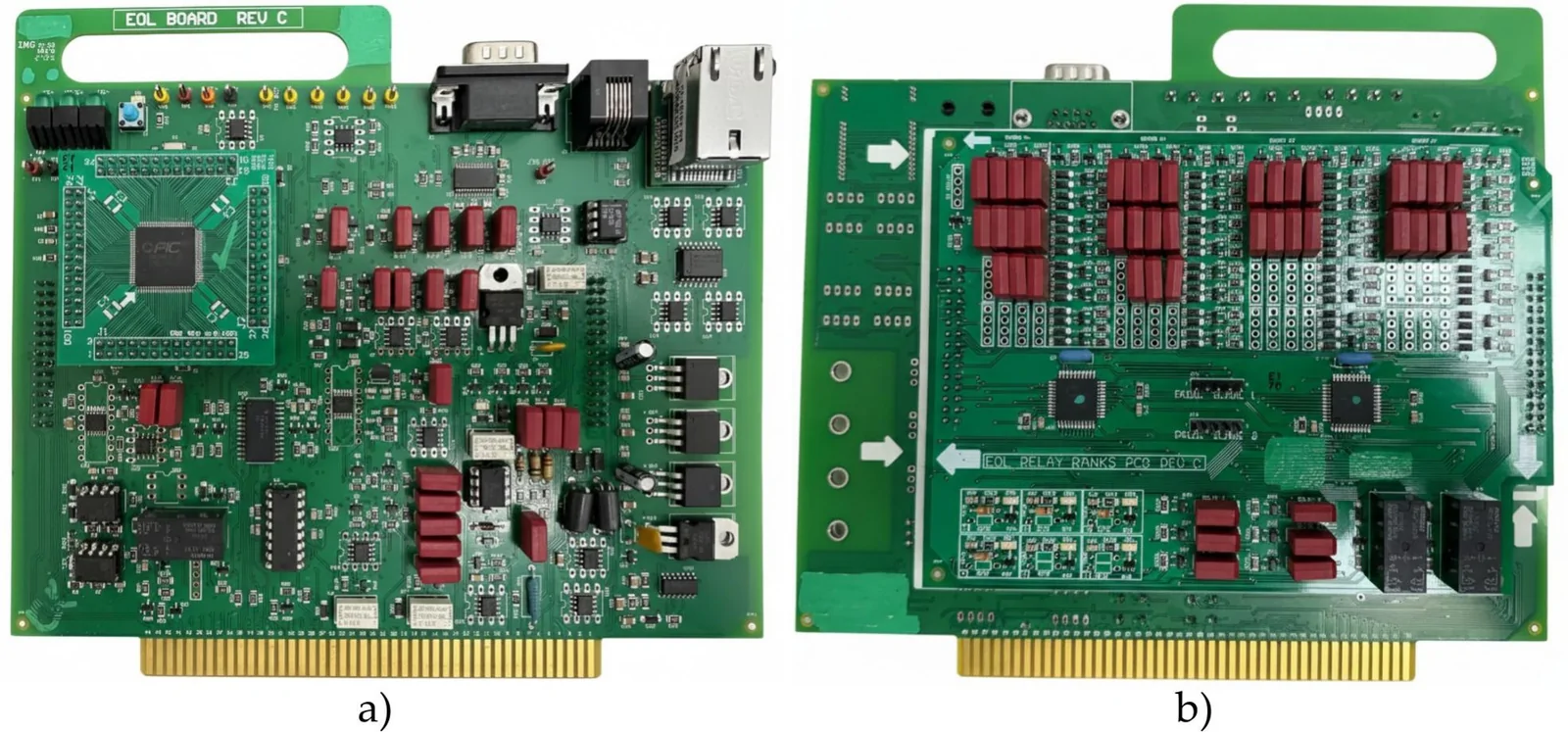
Modular PCB assembly. (a) Top side, and (b) bottom side.

The physical implementation of the developed modular PCB assembly, the Electrical Testing Board, is shown in Fig. 7. The mechanical topography features an integrated structural handle along the upper profile and a gold-finger edge connector at the lower boundary optimized for high-density backplane bus integration, constraining the total footprint to 187 × 160 mm.

To balance routing density with on-site serviceability, the board assembly employs a mixed-technology framework with a 70 % Surface-Mount Technology (SMT) and 30 % Through-Hole Technology (THT) distribution. The SMT allocation accommodates high-density digital routing, the microcontroller unit (MCU), and high-fidelity signal-conditioning topologies. Passives are standardized to 1206 packages, while active semiconductors utilize Small Outline Integrated Circuit (SOIC) and Thin Shrink Small Outline Package (TSSOP) geometries. This packaging strategy optimizes spatial efficiency while allowing manual rework. Conversely, the THT allocation is reserved for electromechanical relays and ruggedized connectors, leveraging their mechanical robustness and fatigue resistance under cyclic operational stress.

Furthermore, mission-critical integrated circuits are provisioned in Dual In-line Packages (DIP) and hosted within dedicated sockets. This hybrid manufacturing approach yields a highly resilient, modular framework that mitigates maintenance overhead by facilitating rapid, localized component replacement throughout the platform’s operational lifecycle.

### 6.2 PCBs construction

#### 6.2.1 Considerations

**Fig. 8:**
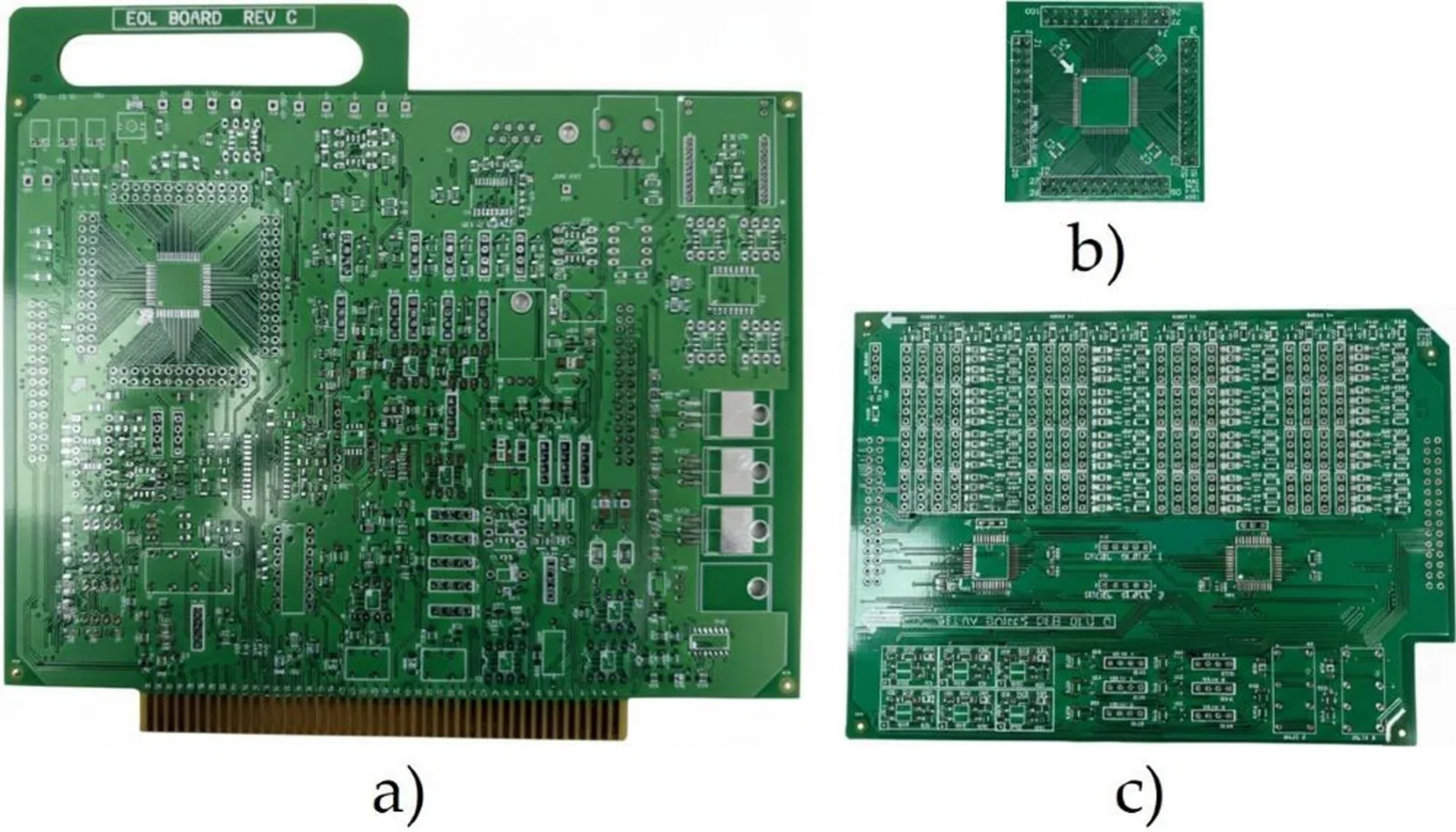
Electrical testing board. (a) Main PCB, (b) Relays PCB, and (c) MCU PCB.

The construction process of the developed modular PCB assembly involves several considerations, including safety precautions and specialized tools. First, as a safety consideration, manually populating electronic components onto the PCBs shown in Fig. 8 requires high-temperature soldering and must be handled with care to prevent burns. Tools such as soldering iron stations for through-hole components and reflow soldering stations for SMD are needed. Tools must be used with care in a well-ventilated environment, as soldering may contain toxic materials.

Alternatively, PCBs can be populated automatically with SMT equipment for surface-mount device placement. This is achieved by building mechanical stencils from the design files specified in Table 8, which are then used to dispense solder paste onto the PCBs and to populate them with automatic pick-and-place equipment. The soldering process then proceeds directly to reflow ovens for the final process. Additionally, through-hole components can be soldered by building a mechanical fixture for use with industrial wave soldering equipment. Additionally, to maximize flexibility in component sourcing, an adaptive hardware approach was adopted: a hybrid land pattern for certain integrated-circuit components, meaning the designed footprints accommodate both SOIC and TSSOP SMDs and DIP through-hole packages, allowing seamless assembly regardless of package availability.

#### 6.2.2 Construction steps

The construction steps should be followed as described below, regardless of whether electronic components are populated manually or with automatic placement equipment.

1) Core MCU Sub-Board Assembly (MCU PCB)

1.1 Monolithic IC Placement: The primary MCU (PIC32MX795F512L) must be precision-aligned and reflowed or soldered onto the dedicated MCU daughterboard. This sub-board is architected as a modular, hot-swappable mezzanine card to optimize localized field serviceability.

1.2 Interconnect Fabrication: Following IC integration, low-profile, through-hole female socket headers are soldered to the perimeter pads, completing the pluggable MCU sub-assembly.

2) Main PCB

2.1 Thermal Profile Management (SMT first): All SMT components must undergo initial deposition and soldering. This sequence prevents localized thermal degradation of subsequent plastic-housed electromechanical components during thermal processing.

2.2 Polarized Component Orientation: Electromechanical relays must be populated in compliance with the spatial orientations designated by the silkscreen fiducials.

2.3 Socketed Silicon Architecture: DIP integrated circuits must not be subjected to direct soldering. Instead, low-contact-resistance DIP sockets must be soldered to the PCB matrix to facilitate non-destructive IC replacement.

2.4 Ruggedized Parasitic and Connector Population: The remaining THT array—encompassing pitch headers, communication interfaces, polarized electrolytic decoupling capacitors, ferrite beads, PolySwitch resettable fuses, and dedicated test points—is populated and wave- or hand-soldered.

2.5 Post-Assembly Metrological Inspection: To ensure structural integrity, a high-magnification optical inspection must be performed across all solder junctions to identify and rectify bridging defects or cold joints before subsystem powering.

3) Relay Board Sub-Assembly (Relay PCB)

3.1 Primary SMT Phase: Surface-mount components are processed during the initial assembly phase to mitigate thermal shock risks to downstream mechanical components.

3.2 Relay Array Alignment: Electromechanical relays are aligned and soldered according to the explicit silkscreen orientation matrices.

3.3 Peripheral THT Integration: Peripheral through-hole elements, including localized pin headers, are systematically populated and secured.

3.4 Quality Assurance Optical Verification: Microscopic or optical inspection is executed across the entire interconnect profile to eliminate latent short-circuits before validating electrical functionality.

#### 6.2.3 PCBs assembly steps

**Fig. 9:**
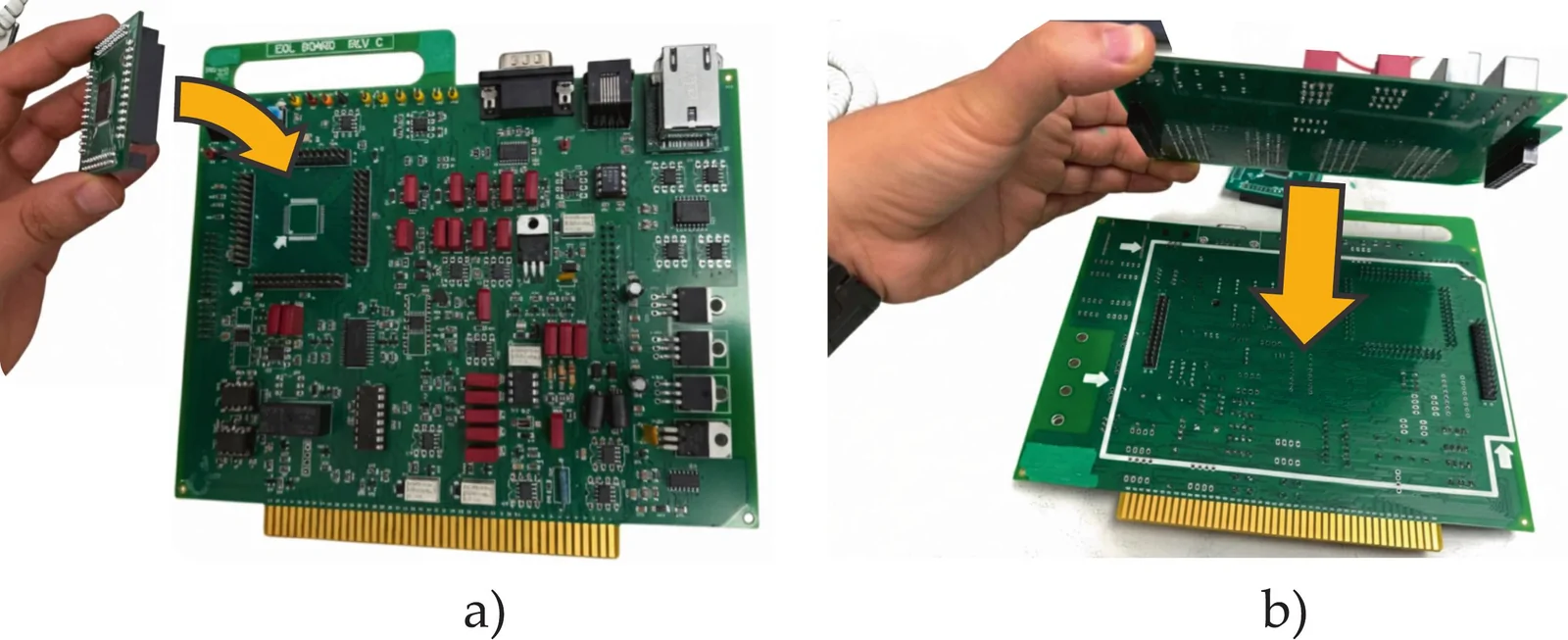
Assembly process. (a) PCB MCU assembly to the Main PCB, and (b) Relay PCB assembly to the Main PCB.

Following substrate fabrication, system integration relies on a direct-mating interconnect scheme utilizing paired male and female pin-header matrices. To mitigate the risks of polarity inversion or mechanical misalignment, the assembly workflow utilizes explicit geometric keys and orientation fiducials defined on the PCB silkscreen layers (Fig. 9). The integration sequence initiates with the mechanical and electrical coupling of the MCU PCB card to the primary Main PCB. This phase requires strict registration of the alignment arrows on the left-hand silkscreen profiles of both substrates; precise orientation synchronization is mandatory to prevent terminal electrical overstress or catastrophic system failure. The final integration phase involves mating the Relay PCB to the bottom layer of the “Main PCB” architecture, aligning with the vectors indicated by the corresponding silkscreen orientation arrows.

#### 6.2.4 Power-up modular PCB assembly

**Fig. 10:**
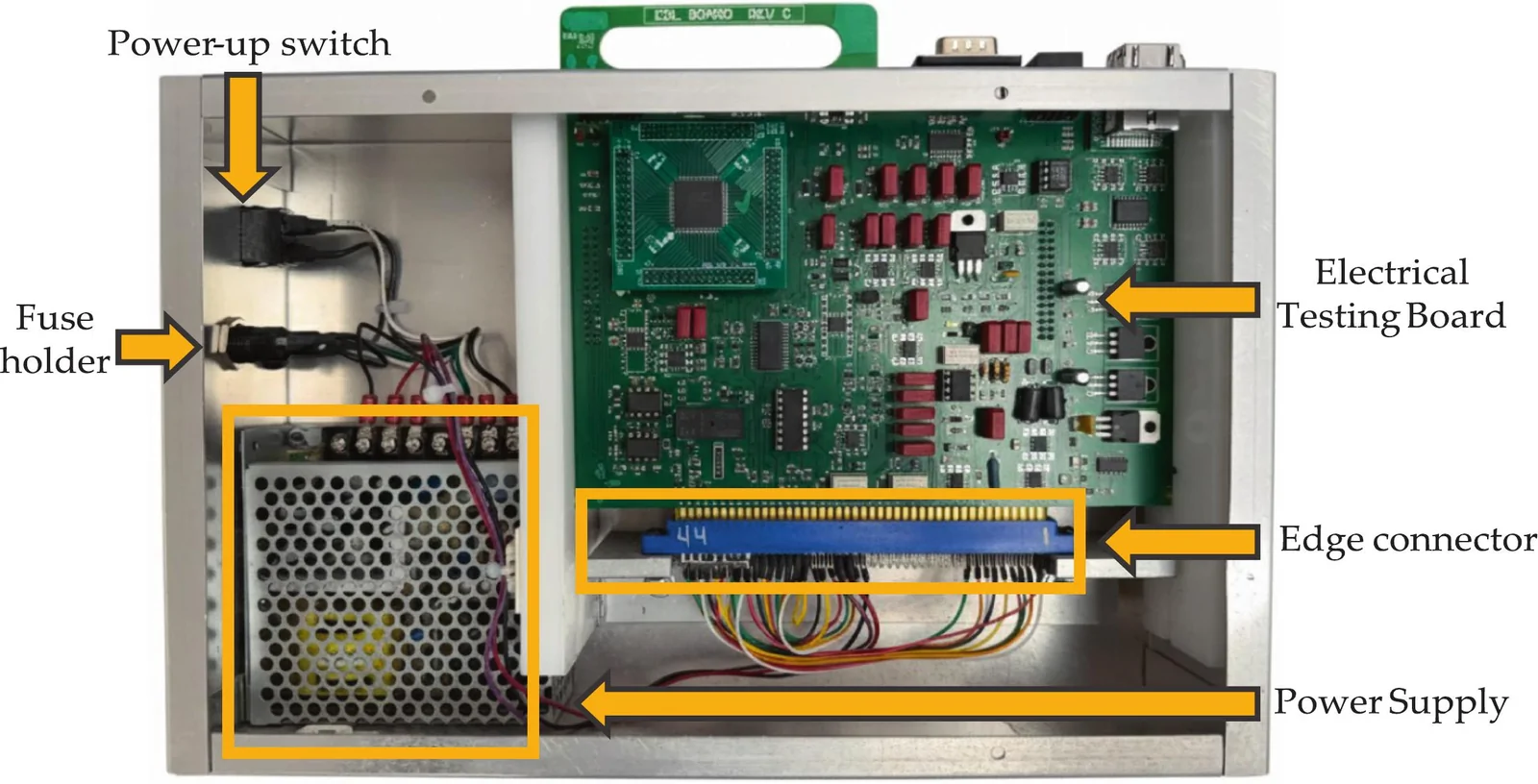
Diagnostics and debugging enclosure.

The modular PCB assembly requires a power supply system capable of delivering high-precision ±15 V bipolar outputs to drive the onboard instrumentation; these rails are supplied directly to the Main PCB gold-finger edge connector (DigiKey PN: ECC44DREH-S13-ND). From an operational safety perspective, handling this dual-rail power infrastructure requires strict adherence to electrical safety precautions by the end user. Because the edge connector lacks hardware-level reverse-polarity protection to preserve signal integrity, the user must verify terminal orientation and ensure symmetric voltage-power sequencing before energizing the system. Asymmetrical rail initialization or floating-ground conditions pose a severe risk of localized electrical overstress, potentially inducing latch-up or catastrophic thermal degradation in sensitive analog instrumentation nodes. Furthermore, manual diagnostic profiling must be restricted to insulated tools to prevent accidental short-circuits or the injection of electrostatic discharge (ESD) into high-impedance measurement paths. To mitigate these electrical hazards, the non-conductive enclosure (DigiKey PN: HM2732-ND) shown in Fig. 10 functions as the primary physical isolation barrier, preventing accidental contact with energized traces and establishing structural environmental shielding during bench-top testing. Table 8 lists the corresponding manufacturing files required to build this enclosure.

## 7 Operation instructions

### 7.1 Initialization

Prior to system deployment, the main MCU—a PIC32MX795F512L—and both secondary PIC18F4620 slave MCUs require firmware provisioning to establish the central control logic and auxiliary task-execution routines.

**Fig. 11:**
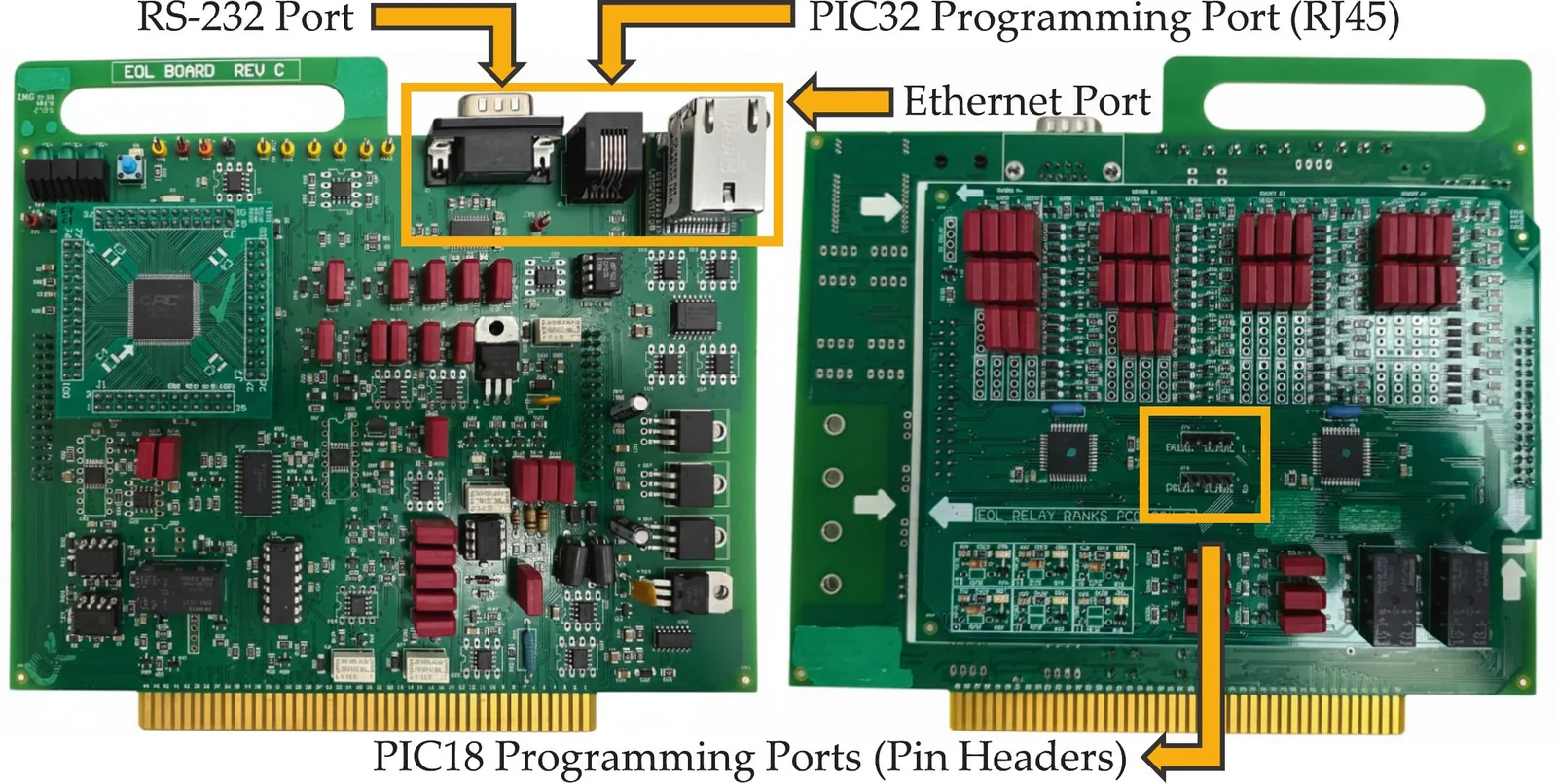
Connector ports.
2) The PIC28F4620 MCUs' firmware is programmed only once; these devices act as slave devices. Programming access is via female pin headers near both MCUs, as shown in Fig. 11. Note that the firmware is identical across both devices. Each slave MCU has a hardware address via GPIOs, allowing access to the firmware to handle specific tasks.

1) The PIC32MX795F512L MCU firmware must be programmed via the RJ45 connector attached to the Main Board, as shown in Fig. 11.

Firmware deployment is executed via an MPLAB ICD 5 in-circuit debugger/programmer, though alternative Microchip hardware architectures—such as the PICkit series or legacy ICD variants—remain compatible with the targeted PIC18 and PIC32 MCU families. Toolchain synthesis requires the designated Microchip C18 (v3.46) and XC32 (v4.60) compiler versions or later, which are available for free from the official Microchip repository.

The firmware flashing sequence can be performed using either the host Electrical Testing Board’s internal power rail or the hardware programmer’s internal power supply; this setting is explicitly managed in the power configuration parameters of the MPLAB X IDE. Crucially, the end user must ensure that the external programmer’s supply and the modular PCB assembly’s power infrastructure are never energized simultaneously. Dual-source power collisions eliminate isolation boundaries and introduce severe risks of conflicting supply rails, leading to catastrophic electrical overstress and irreversible damage to MCU cores.

Firmware deployment is executed via an MPLAB ICD 5 in-circuit debugger/programmer, though alternative Microchip hardware architectures—such as the PICkit series or legacy ICD variants—remain compatible with the targeted PIC18 and PIC32 MCU families. Toolchain synthesis requires the designated Microchip C18 (v3.46) and XC32 (v4.60) compiler versions or later, which are available for free from the official Microchip repository.

The firmware flashing sequence can be performed using either the host Electrical Testing Board’s internal power rail or the hardware programmer’s internal power supply; this setting is explicitly managed in the power configuration parameters of the MPLAB X IDE. Crucially, the end user must ensure that the external programmer’s supply and the modular PCB assembly’s power infrastructure are never energized simultaneously. Dual-source power collisions eliminate isolation boundaries and introduce severe risks of supply rail conflicts, leading to catastrophic electrical overstress and irreversible damage to MCU cores.

### 7.2 Suggested implementation overview

**Fig. 12:**
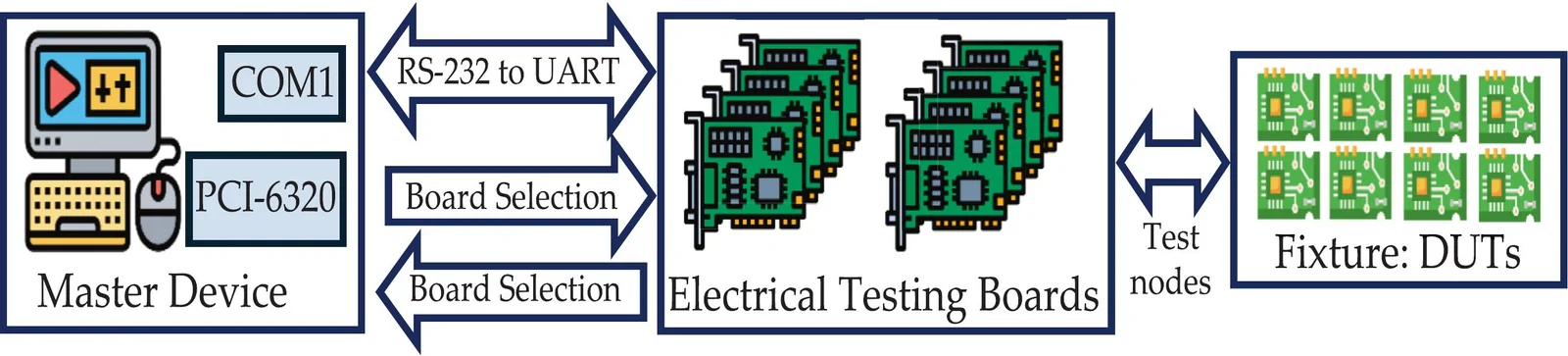
Modular PCB assembly implementation overview.

The architecture of the developed modular PCB assembly is engineered primarily for integration into Automated Test Equipment (ATE) systems that execute simultaneous end-of-line functional and in-circuit parallel testing. This operational framework is realized via a customized test fixture configured with independent nests for the DUT. By dedicating an individual modular PCB assembly to each discrete nest, the system enables independent, concurrent, parallel test sequences, as shown in the schematic topology of Fig. 12.

#### 7.2.1 Control

System orchestration is governed via RS-232 serial communication protocol, with a central host computer (PC) acting as the host controller. The host interfaces through a native COM1 port, where the RS-232 signal levels are translated to logic-level UART to establish direct parallel control connectivity with the opto-isolated UART module embedded within the board’s gold-finger edge connector. Concurrently, a National Instruments (NI) PCI-6320 Data Acquisition (DAQ) card is integrated into the host controller to manage the digital I/O lines. This hardware drives the digital outputs for board-selection routing and monitors the digital inputs for the output board’s busy-status flags. To facilitate multi-board orchestration, the control architecture implements two primary discrete signaling lines:

1) Board-selection. A dedicated digital input line that enables a specific testing board to decode and execute incoming RS-232 instructions.
2) Busy-status output: A digital output signaling real-time board operational availability to the host controller.

Through this topology, the host controller manages multiple parallel modular PCB assemblies via discrete digital output selection. This configuration establishes a hardware-level media access control addressing scheme, as all interconnected board nodes concurrently receive broadcast RS-232 data packets over the COM1 port but discard them unless explicitly addressed.

The sequential control protocol and packet-transmission architecture executed between the host controller and the modular PCB assembly during an automated testing cycle are structured as follows:

1. Hardware Board Selection: The master host asserts a logical low-to-high transition on a dedicated digital output line of the NI PCI-6320 DAQ card, enabling the addressing matrix of the target modular PCB assembly.
2. Communication process, parameter, and command provisioning: The communication operates in a deterministic duplex sequence: (i) the host broadcasts a structured configuration packet containing the DUT profile, precise electrical test limits, tolerances, and cross-point matrix node mappings; (ii) the host subsequently issues an execution command to initialize the automated firmware testing routine based on the pre-loaded parameters.
3. Board busy status: Upon parsing the execution command, the local MCU drives its busy-status digital output from a low to a high logic level, signaling real-time task execution to the host controller. The host controller executes a synchronous polling routine, monitoring this flag until the board assembly completes the test sequence and pulls the busy line low, signaling cycle completion.
4. Data Acquisition and Extraction: Upon detecting the negative-edge transition of the busy flag, the primary node transmits a specialized request-result packet over the serial bus to retrieve the captured parametric data from the board’s internal buffers.

To maximize throughput, the local modular PCB assembly processes these instructions via an optimized hardware loop at a standardized baud rate of 115,200 bps, minimizing latency overhead during back-to-back test cycles.

#### 7.2.2 Testing tasks

The modular PCB assembly executes parametric and functional evaluations tailored to the specific requirements of a DUT. As summarized in Table 2, the board’s operational capabilities are augmented by relay-based switching matrices that enable dynamic configurations for both ICT and FCT topologies. Implementing this platform during an automated end-of-line testing phase establishes a critical final quality-assurance gateway for validating system functionality before product packaging. Some typical capabilities supported by the PCB assembly might include:

1. Continuity and Isolation Profiling: Verification of wire paths and harness integrity via short-circuit and open-circuit isolation testing.
2. Dynamic Stimulus-Response Characterization: Application of precise voltage stimuli to the DUT coupled with synchronized response monitoring of analog signal-conditioning chains.
3. Parametric Volumetric and Current Metering: High-resolution voltage measurements and ammeter-based monitoring of quiescent and standby currents.
4. Impedance Metrology: Passive component characterization through resistance and capacitance extraction.
5. Mixed-Signal Data Acquisition: Simultaneous capture and digitization of analog and digital signal lines.
6. Acoustic Transduction and Verification: Audio-frequency signal detection and spectral validation.
7. Bus Telemetry and Non-Volatile Provisioning: Bidirectional digital communication over UART, I^2^C, or SPI protocols to read firmware registers or write factory-calibration parameters into a DUT’s non-volatile memory.
8. Arbitrary Mixed-Signal Routing: Reconfigurable routing of complex input/output signals tailored to custom functional test vectors.

## 8 Validation and characterization

**Fig. 13:**
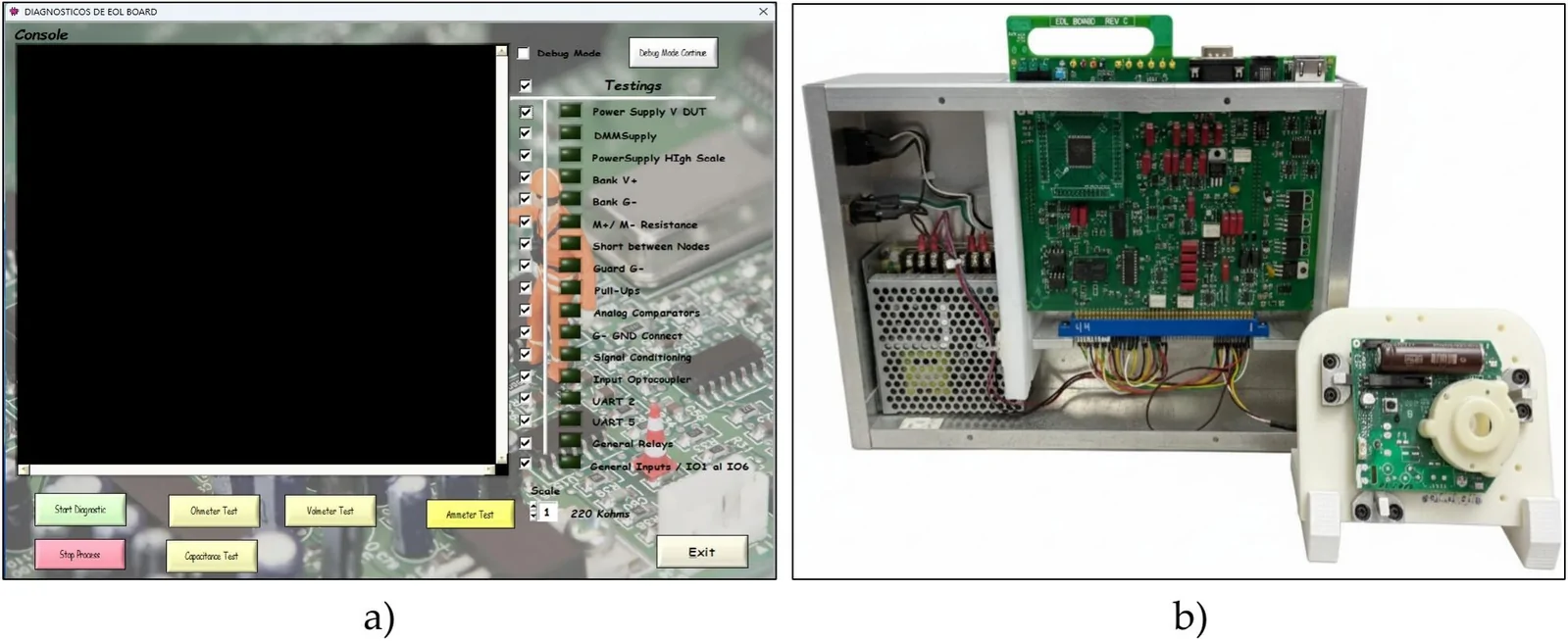
(a) Graphical user interface and (b) modular PCB assembly with a DUT.

The experimental evaluation focuses on the functional validation of the integrated modular PCB assembly hardware and firmware architecture (shown in Fig. 13), as well as its empirical validation within a multi-site ATE framework. The software interface presented in this work was developed solely as an internal hardware-validation GUI to evaluate the modular PCB assembly. Consequently, high-level end-user features such as production pass/fail dashboards, automated reporting, and factory-level MES/SCADA integration were beyond the scope of this work and represent targets for future software development.

### 8.1 Experimental validation

To facilitate comprehensive system validation, a dedicated GUI was developed within the LabWindows/CVI integrated environment. This software layer establishes bidirectional communication with the Modular PCB assembly via the RS-232 serial interface, enabling discrete, modular control and diagnostic monitoring during both in-circuit and functional tests. Fig. 13 shows the deployed GUI alongside the physical deployment of the energized diagnostic and debugging enclosure configured for localized hardware verification. Note: The system architecture supports alternative control methods beyond the standard RS-232 serial interface and LabWindows/CVI GUI presented in this work. Developers can implement custom control layers using Python scripts, LabVIEW VIs, or native C/C++ applications that communicate via alternative hardware interfaces, such as TCP/IP over Ethernet.

These custom interfaces can interact directly with the corresponding data packet structure shown in Fig. 3 for each testing task. The GUI layouts and firmware command sets are fully open-source, user-modifiable, and documented in Table 8.

The empirical validation for the modular PCB assembly is executed systematically across distinct subsystem layers. First, the initialization phase assesses the reliability of the relay-based switching matrices, the regulation stability, and the load-transient response of the programmable power supplies under a designated resistive load across multiple voltage spans. Secondary, the validation routines verify peripheral subsystems, including analog comparators, signal-conditioning blocks, acoustic transduction circuits, software-configurable pull-up/pull-down networks, and opto-isolated UART communication lines. Finally, the instrumentation suite evaluates the system’s parametric in-circuit measurement accuracy. The GUI orchestrates these automated verification routines, analyzing the transfer functions and tolerances of the onboard ohmmeter, capacitance-meter, voltmeter, and ammeter modules while preserving low-level, direct hardware access for active debugging. While the following validation framework evaluates functional performance and short-term measurement repeatability, metrological factors such as thermal sensitivity, long-term calibration drift, and inter-bus noise coupling were not characterized, thereby representing clear system limitations that warrant attention in future work.

Similarly, while the modular assembly incorporates design provisions and foundational safeguards such as optical isolation, overcurrent protection, and ESD suppression diodes, operational robustness under harsh industrial fault conditions—specifically high-voltage ESD events, active DUT fault injection, and incorrect fixture connections—was not experimentally evaluated and represents an additional scope limitation to be addressed in future work.

#### 8.1.1 Relay reliability

Because the relay-based switching matrices serve as the primary interface routing discrete ICT and FCT modules to their respective test nodes, validation is performed for each node on each matrix bus (i.e., V+, M+, M−, and G − ). The switching topologies are constructed using Coto Technology 9012–12-11 reed relays, which exhibit a typical electromechanical actuation time of 0.35 ms, including a 0.1 ms contact-bounce duration. The GUI orchestrates a practical validation procedure via the following sequence:

1. Each test node selection is measured in terms of the relay-based switching matrices for node continuity (short-circuit detection) between nodes, cross-point isolation (open-circuit detection), and relay contact resistance.
2. To evaluate dynamic contact resistance stability and isolate the effects of transient switching noise, the measurement routine is replicated over *N* = 100 continuous iterations per node. A polling interval of 50 ms is maintained between consecutive tests, and a firmware-enforced settling delay of 1.0–2.0 ms is strictly required before triggering the ADC acquisition phase.

The operational longevity and contact reliability figures presented in this work rely on component-level manufacturer specifications—specifically, relay mechanical lifetimes exceeding 10^7^ and electrical lifetimes ranging from 10^5^ to 10^6^ cycles under nominal loads. Due to equipment and time constraints, long-term empirical endurance testing, accelerated life cycling, and quantitative tracking of contact resistance degradation were not performed on the fully integrated platform. Additionally, the maximum load stress testing was not explicitly performed; rather, the power and signal paths were intentionally restricted to operate within the relay’s maximum carry current rating (1.5 A, per manufacturer specifications) to ensure device reliability, while low-voltage and low-current operation inherently mitigates contact arcing, the long-term degradation of the switching matrices under continuous operational stress, as well as the characterization of transient switching noise, remain key system limitations.

Comprehensive empirical reliability validation and MTBF analysis under extended operating conditions are designated as focus areas for future investigation. Accordingly, the proposed validation framework is deployed as a diagnostic tool for failure analysis, facilitating the isolation of faulty or degraded switching matrix components.

#### 8.1.2 Master-Slave MCU architecture under failure conditions

The fault detection and reporting mechanisms of the Master–Slave MCU architecture were evaluated across diverse failure modes. The master MCU (PIC32MX795F512L) was configured to detect and transmit critical operational anomalies to the host controller via the primary data communication interface. Specifically, the system identified and reported data framing errors (e.g., incomplete packet transfers), transmission corruption indicated by cyclic redundancy check (CRC) or checksum mismatches, and specific fault codes generated during ICT/FCT operations. Furthermore, the master MCU exhibited reliable fault logging during real-time exceptions, including communication timeouts, Watchdog Timer (WDT) reset events, and unannounced power interruptions that triggered hardware resets. In parallel, the slave MCUs (PIC18F4620) were configured for local fault detection and reported anomalous conditions directly to the master MCU via the I2C bus, establishing a hierarchical telemetry loop.

Table 9 synthesizes these evaluated failure modes, detailing their operational impacts, detection mechanisms, and corresponding mitigation strategies. Notwithstanding, these integrated software detection mechanisms represent software provisions that help to address some failure scenarios via mitigation strategies rather than automated recovery behavior under severe operational conditions—such as mid-execution signal line disconnection, continuous hardware-level bus noise, or active fault injection into the slave nodes—which were not empirically characterized in this work. In the event of an unrecoverable firmware stall or complete loss of communication, the system relies on manual intervention and power cycling to restore normal operation. Consequently, comprehensive real-time fault recovery under harsh industrial fault scenarios remains an explicit scope limitation to be addressed in future hardware and firmware revisions.

**Table 9:** Failure mode, effects, and mitigation strategies for the Main–Slave MCU architecture.

Failure modes	Operational impacts	Detection mechanisms	Mitigation strategies
Data Framing Error	Incomplete data packets; incomplete message processing.	Protocol layer packet length and boundary verification.	Flag error, drop corrupt packet, and assign a specific failure code to the host controller.
Data Corruption	Mismatched data states	Checksum mismatch during host controller–Master MCU communication.	Reject packet, return error status code, and request packet retransmission.
Communication Timeout	Inter-module responsiveness loss.	Real-time communication timer expiration.	Trigger timeout event, assign error code, and re-initialize bus interface.
ICT / FCT Process Exception	Testing fault; functional step failure	Hardware diagnostic routines and testing timer expiration.	Halt execution, log internal state, and output failure diagnostic code to host controller.
Power-up Interruption	Transient system drop; voltage dip causing power-on reset (POR)	Internal Power-on Reset (POR) at Master MCU.	Executive safe-state reboot; transmit boot status and power fault code to host controller.
Slave MCU I^2^C Fault	Local subsystem error; peripheral processing failure	Interruption handler and communication timeout expiration with Master MCU.	Transmit diagnostic telemetry to Master PIC32MX; execute deterministic local safe mode.

#### 8.1.3 Communication approach

The data transceiver architecture was verified by routing structured packets from the master host across three onboard communication interfaces: the opto-isolated UART buses, the physical RS-232 serial link, and the Ethernet TCP/IP interface. To evaluate the throughput and signal integrity of the physical layer, simultaneous data streams were broadcast at a standardized baud rate of 115,200 bps via the opto-isolated UART pins embedded on the gold-finger edge connector and the dedicated RS-232 DB9 interface; this validated real-time UART packet processing and interrupt handling within the primary PIC32MX795F512L MCU. Concurrently, the Ethernet capabilities were characterized by establishing a direct connection between the hardware platform and a local intranet. This configuration verified remote network accessibility and sockets-based communication via a statically assigned IP address. Each implemented communication protocol provides deterministic, low-level abstraction layers that enable direct control of the platform and offer a versatile telemetry framework for executing complex, multi-site automated testing tasks tailored to the deployment strategy.

#### 8.1.4 Programmable power supplies

The modular PCB assembly features four independent programmable power supplies tailored for specific hardware functions: one rail dedicated to parametric current monitoring, one for cross-reference voltage stabilization, and two configured exclusively for dynamic voltage stimuli. Experimental validation focused on characterizing the DAQ_1 and DMM_Supply stimuli rails across a 0 V to 10 V dynamic operating range under both no-load (open-circuit) and loaded conditions. The transfer function calibration was performed from a true 0 V baseline to the 10 V full-scale limit using discrete 50 mV increments (Δ*V* = 50 mV). Output voltages were monitored using a high-precision HP 34401A 6½-digit digital multimeter. To rigorously assess measurement uncertainty and repeatability, a statistical analysis was performed across *N* = 100 independent sample sets.

The transfer function exhibited high linearity across the full dynamic span, a characteristic directly attributable to the integration of a low-drift, 10 V precision voltage reference. Type A evaluation of standard uncertainty revealed a combined experimental standard deviation of σ = ±14 mV across the entire voltage range, demonstrating excellent reproducibility and tight error-bounding under repetitive test cycles. Furthermore, high-current load regulation was evaluated by applying a continuous 1 A load step to the output. The rail maintained high-voltage stability, confirming the efficacy of the active pre-regulator circuit architecture, which is designed to minimize thermal dissipation across the linear regulation stages while sourcing up to 1 A. This robust driving capability successfully validated the hybrid firmware-hardware overcurrent protection (OCP) network. By utilizing the software-controlled CURRENT_PROG parameter, current-limit thresholds can be dynamically mapped across distinct operational ranges to ensure deterministic electrical overstress protection. This firmware-configured analog control voltage maps linearly to a physical overcurrent threshold at a ratio of 5 V/1 A (5 mV/mA). For instance, a CURRENT_PROG output of 5 V establishes a 1 A current-consumption boundary; exceeding this threshold triggers a hardware comparator that latches the overcurrent protection circuit into a safe state. Additionally, the CROSS_REF_VOLT rail was verified to deliver an exceptionally stable reference potential, guaranteeing cross-module measurement coherence and minimizing differential gain errors across all interconnected analog subsystems.

#### 8.1.5 DMM: voltmeter

The acquisition accuracy of the onboard voltmeter was evaluated under real-time operating conditions within a continuous, automated testing loop. Dynamic voltages were injected using a high-stability Keysight N7900 programmable power supply, which interfaced directly with the system’s high-sensitivity M+ (Measurement Positive) bus to Node 1 and G−bus (Guarded Ground) to Node 2 via the specialized diagnostics and debugging enclosure. An HP 34401A 6½-digit digital multimeter was used as a reference instrument to compare data. Telemetry extraction was monitored in real time through the GUI to quantify measurement uncertainty and system repeatability rigorously. Table 10 outlines the statistical evaluation at six measurement points for the low-voltage range (0 V to 10 V) and high-voltage range (0 V to 100 V); each measurement point was conducted over *N* = 100 independent measurements across the full operational span.

**Table 10:** Voltmeter parametric validation and statistical error profile of the modular PCB assembly.

Measurement point	HP 34401A	Modular PCB assembly				
	Measured reference value	Mean value	Absolute error	Relative error %	σ	CI 95 %
1.0 V	0.99998 V	0.9985 V	0.001381 V	0.138 %	616 µV	[0.99849, 0.99874] V
3.3 V	3.30051 V	3.2989 V	0.002385 V	0.072 %	2.734 mV	[3.29837, 3.29946] V
5.0 V	4.99985 V	5.0038 V	0.003884 V	0.077 %	1.4 mV	[5.00360, 5.00416] V
9.0 V	9.00015 V	9.0435 V	0.043516 V	0.483 %	43 mV	[9.03496, 9.05207] V
30.0 V	30.0005 V	30.158 V	0.1584 V	0.528 %	62.72 mV	[30.1456, 30.1713] V
90.0 V	89.9985 V	94.245 V	0.86711 V	0.9127 %	633 mV	[94.1171, 94.3684] V

The comparative validation of the modular PCB assembly against the HP 34401A baseline reveals a clear correlation between the nominal voltage tier and measurement divergence. Low-voltage power domains (≤5 V) exhibited highly precise regulation profiles, characterized by narrow 95 % confidence intervals and maximum relative error rates strictly bound below 0.14 %. Conversely, higher-potential planes showed a pronounced increase in both systemic baseline offsets and signal variance. This performance degradation is most pronounced at the 90.0 V measurement tier, where the assembly operated at a substantial positive mean offset of 94.245 V, a heightened relative error of 0.9127 %, and an elevated standard deviation σ = 633 mV. This progressive variance scaling indicates increased susceptibility within the higher-voltage rails to parasitic loading effects, thermal fluctuations, or localized component tolerances in the integration of the shared power planes. Additionally, uncertainty is directly related to the attenuation factor of the internal scaling resistor networks required to bring the 100 V transients within the safe operating limits of the MCU’s ADCs.

#### 8.1.6 DMM: ammeter

The accuracy of the onboard parametric current acquisition ammeter was empirically evaluated under real-time operating conditions within a continuous, automated testing loop. Dynamic current profiles were injected using a high-stability Keysight N7900 programmable power supply interfaced to Node 1 via the system’s G− bus. Node 1 connects to a Transimpedance Amplifier (TIA) circuit that maps the input current to a proportional analog voltage measured into the M+ buffer. A forward-biased signal diode (*V*_forward_) is integrated into the topology to establish a stable biasing threshold and provide overcurrent protection for the instrumentation loop within the diagnostics enclosure. For metrological verification, an HP 34401A 6½-digit digital multimeter served as the reference standard. In-series current measurements were obtained by opening jumper J9 on the modular PCB assembly to insert the reference DMM directly into the signal path. Parametric current acquisition is executed via a software-configurable, three-scale ammeter network. To rigorously characterize the precision and repeatability of these current-sensing topologies, a statistical evaluation was performed using N = 100 independent measurements per configuration. Table 11 outlines the statistical evaluation across nine representative measurement points: Micro-Current Scale I (1 µA to 10 µA), Micro-Current Scale II (10 µA to 100 µA), and Milli-Current Scale III (0.1 mA to 1.0 mA).

**Table 11:** Ammeter parametric validation and statistical error profile of the modular PCB assembly.

Scale		Measurement point		HP 34401A		Modular PCB assembly								
				Measured reference value		Mean value		Absolute error		Relative error %		σ		CI 95 %
I		1 µA		1.01 µA		1.021 µA		10.2nA		1.02 %		5.10nA		[1.009, 1.011] µA
		5 µA		5.006 µA		4.997 µA		2.32nA		0.046 %		192nA		[4.95, 5.036] µA
		10 µA		10.003 µA		10.07 µA		75.5nA		0.75 %		320nA		[10.01, 10.14] µA
II		15 µA		15.0061µA		15.02 µA		27.6nA		0.18 %		698nA		[14.88, 15.16] µA
		50 µA		50.001 µA		50.9 µA		933nA		1.86 %		1.52 µA		[50.63, 51.23] µA
		100 µA		99.987 µA		106.81 µA		6.94 µA		6.81 %		11.87 µA		[104.4, 109.1] µA
III		150 µA		150.056 µA		149.97 µA		27.68nA		0.018 %		14.47 µA		[147.09, 152.84] µA
		500 µA		500.0065 µA		505.17 µA		5.17 µA		1.03 %		21.10µA		[500.98, 509.35] µA
	1 mA		1.0005 mA		1.051 mA		51.59 µA		5.15 %		37.62 µA		[1.044, 1.059] mA	

Quantitative metrological comparison between the standard reference measurements obtained with the HP 34401A digital multimeter and the proposed modular PCB assembly across three distinct measurement ranges (I, II, and III). Overall, the custom acquisition system demonstrates close agreement with the benchmark instrumentation, achieving sub-percent relative errors across low-level current test points such as 5 µA (−0.046 %) and 150 µA (0.018 %). Although minor systematic deviations and broader dispersion (σ) are observed at the upper limits of each sub-range—most notably at 100 µA (6.81 % relative error) and 1 mA (5.15 % relative error)—the confidence intervals (95 % CI) confirm consistent system stability and operational reliability for low-cost functional testing applications.

#### 8.1.7 DMM: ohmmeter and capacitance meter

The acquisition accuracy of the onboard ohmmeter was evaluated under real-time operating conditions within a continuous, automated testing loop using the specialized diagnostic enclosure in conjunction with the system’s GUI. To establish metrological traceability and characterize system repeatability, the validation protocol was executed using high-tolerance reference standards—specifically, axial-leaded resistors spanning 0.5 Ω to 1 MΩ with 0.1 % tolerance. For metrological verification, an HP 34401A 6½-digit digital multimeter served as the reference standard. The resistors were directly interfaced with the high-sensitivity sensing channels by connecting the R_DUT_ terminals via the diagnostic enclosure, routing V+ (R_DUT_ axial terminal 1) to Node 1 and M− (R_DUT_ axial terminal 2) to Node 2, and then directly to the software-programmable pull-downs module. Table 12 outlines the statistical evaluation performed over *N* = 100 independent measurements per passive component tier across five representative measurement points spanning the full operational range.

**Table 12:** Ohmmeter parametric validation and statistical error profile of the modular PCB assembly.

Measurement point	HP 34401A	Modular PCB assembly				
	Measured reference value	Mean value	Absolute error	Relative error %	σ	CI 95 %
0.5 Ω	0.5007 Ω	0.556 Ω	56.5 mΩ	11.31 %	4.64 mΩ	[547.3, 565.7] mΩ
1 kΩ	1.00004 kΩ	999.29 Ω	702.3 mΩ	0.070 %	5.42 Ω	[998.22, 1000.37] Ω
10 kΩ	10.0195 kΩ	10.0117 kΩ	11.70 Ω	11.70 %	1.20 Ω	[10.0114, 10.0119] kΩ
100 kΩ	99.906 kΩ	99.83 kΩ	0.167 kΩ	−0.167 %	52.3 Ω	[99.827, 99.848] kΩ
1 MΩ	1.0084 MΩ	0.9903 MΩ	9627 Ω	0.9627 %	536 Ω	[0.9902, 0.9904] MΩ

Experimental validation of the modular PCB assembly revealed high measurement fidelity and narrow confidence intervals across the high-resistance range of the impedance spectrum (1 kΩ to 1 MΩ), with relative percentage errors remaining consistently below 1 %. Conversely, the low-to-medium resistance range regime (0.5 Ω) exhibited an elevated relative error of 11.31 % (mean = 0.556 Ω, σ = 4.64 mΩ), a systematic deviation primarily attributable to parasitic trace impedances, relay contact resistances, and interconnect routing inherent to two-wire sensing architectures, which is compensated via an offset value.

Concurrently, the acquisition accuracy of the onboard capacitance meter was evaluated within a continuous, automated testing loop using the specialized diagnostic enclosure in conjunction with the system’s GUI. To establish metrological traceability and characterize system repeatability, the validation protocol was executed using ceramic and electrolytic capacitors spanning 10 nF to 220 µF with 10 % tolerance. For metrological verification, despite the absence of a facility-standard industrial benchtop DMM, a Fluke 289 true-RMS multimeter was utilized as the reference standard. The capacitors were directly interfaced with the high-sensitivity sensing channels by connecting the *C*_DUT_ terminals via the diagnostic enclosure, routing V+ (*C*_DUT_ terminal 1) to Node 1 and M− (*C*_DUT_ terminal 2) to Node 2 directly to the software-programmable pull-downs module and comparators module as shown in Fig. 6. Table 13 outlines the statistical evaluation performed over *N* = 100 independent measurements per passive component tier across four representative measurement points spanning the full operational range.

**Table 13:** Capacitance meter parametric validation and statistical error profile of the modular PCB assembly.

Measurement point	Fluke 289	Modular PCB assembly				
	Measured reference value	Mean value	Absolute error	Relative error %	σ	CI 95 %
10 nF	11.014 nF	13.09 nF	3.09 nF	30.90 %	1.05 nF	[12.759, 13.422] nF
0.1 µF	0.1083 µF	0.124 µF	24.15 nF	24.1 %	10.4 nF	[120.83, 127.46] nF
100 µF	104.18 µF	108.03 µF	8.003 µF	8 %	133.9 nF	[107.9, 108.04] µF
220 µF	216.96 µF	209.3 µF	10.07 µF	10 %	189.6 nF	[208.73, 209.6] µF

The experimental validation of the modular PCB assembly’s integrated capacitance meter against the Fluke 289 baseline highlights a clear inverse relationship between nominal capacitance magnitude and measurement error. At Micro-Farad Range I boundaries (10 nF and 0.1 µF), the system exhibits significant positive measurement divergence, with relative errors peaking at 30.90 % and 24.1 %, respectively, alongside wider confidence bounds. Conversely, accuracy significantly stabilizes within the Micro-Farad Range II ranges (100 µF and 220 µF), where relative errors remain tightly bounded at or below 10 %. Furthermore, the 100 µF rail demonstrates the highest measurement stability with an exceptionally low standard deviation σ = 133.9 nF, whereas the 220 µF tier shifts to a net negative measurement offset of 209.3 µF. This non-linear scaling across the measurement domains suggests that while the internal instrumentation is highly optimized for larger bulk decoupling reservoirs, parasitic track capacitance or charging-time constraints distort accuracy at smaller picofarad and lower nanofarad thresholds. This progressive scaling of measurement uncertainty in the upper resistance and capacitance tiers is a mathematically predictable consequence of parasitic trace capacitances, leakage currents within the relay routing matrix, and the decreased signal-to-noise ratio (SNR) encountered when measuring low-level charging currents and high-value source impedances.

The integration of resistance and capacitance metrology within this modular PCB assembly is specifically engineered to deliver a highly cost-effective alternative to traditional, high-overhead benchtop instrumentation. By embedding core data-acquisition elements directly onto the board layout, the architecture circumvents the steep capital requirements typically associated with industrial-grade measurement systems. This optimized topology provides an accessible testing framework tailored for controlled laboratory exploration, iterative engineering prototyping, and low-volume production environments.

#### 8.1.8 Programmable pull-ups, pull-downs, and analog comparators

The functional integrity of the programmable pull-up/pull-down networks and analog comparators was evaluated utilizing the GUI and the diagnostic enclosure configuration detailed in Fig. 13b. This specialized verification routing establishes direct physical connections between the internal analog buses and the onboard voltmeter module. Designed explicitly for ICT, these modular sub-circuits enable low-latency open- and short-circuit isolation profiling, as well as localized signal conditioning. Consequently, this architecture provides a highly cost-effective, deterministic hardware solution capable of executing high-speed diagnostic routines at the physical layer. Validation was performed directly across the V+, M+, and M − buses. To characterize the analog comparators, the onboard programmable power supplies were modulated to apply precise voltage stimuli across the input nodes, enabling real-time verification of the comparator switching responses. Concurrently, the programmable pull-up and pull-down resistor networks were validated using the integrated digital multimeter (DMM) module, operating in voltmeter mode to measure and log the resulting node voltages under varying logic states.

#### 8.1.9 Signal amplification, audio conditioning, and multiplexing

The specialized diagnostics and debugging enclosure evaluated analog signal processing in the amplitude domain by interfacing directly with the system buses to route and condition signals for amplification, audio processing, and multiplexed distribution to designated test nodes. To characterize the signal amplification module, a calibrated 0.5 V analog input was injected into the stage. The programmable gain amplifier (PGA) was sequentially configured for nominal voltage gain factors of A*_v_* = 2, A*_v_* = 4, and A*_v_* = 6; the resulting steady-state output voltages were monitored and logged via the onboard voltmeter module to verify linearity and gain accuracy. Concurrently, the acoustic signal-conditioning chain was validated using a standardized, time-varying vocal waveform profile. The analog front-end filtered and amplified the acoustic signal to optimize the dynamic range before quantization. This signal was subsequently digitized into a discrete format, enabling the embedded architecture to perform real-time acoustic pattern integrity analysis and validation routines, such as speech and deterministic pattern recognition.

#### 8.1.10 General opto-isolated inputs, speed-up signal, and EEPROM

The isolation capability of the general-purpose opto-isolated input network was characterized utilizing the diagnostics and debugging enclosure under an external high-voltage stimulus. This architecture ensures complete electrical isolation between the external signal source and the Modular PCB assembly’s internal circuitry. This topology provides a crucial protection layer when interfacing with high-voltage transients or mismatched ground planes, effectively preventing catastrophic ground-loop currents and hardware degradation.

Concurrently, a high-speed Pulse-Width Modulation (PWM) output channel configured with a programmable duty cycle was evaluated. The transient timing parameters were benchmarked using a Keysight DSOX3014A digital storage oscilloscope. The base frequency and resolution of the PWM waveform are determined by the primary system clock of the PIC32MX795F512L MCU, which features a software-configurable phase-locked loop (PLL) that can scale the core operating frequency from 8 MHz to 80 MHz to achieve precise ON and OFF duty cycles.

Finally, the non-volatile memory layer was verified via an independent EEPROM read-and-write validation cycle. Host-configured test limits were transmitted from the control host PC, committed to the EEPROM registers, and subsequently read back to verify the integrity of byte allocation. This non-volatile storage architecture serves as a deterministic repository for retaining critical manufacturing test limits, runtime hardware configurations, and offset calibration coefficients.

#### 8.1.11 General-purpose inputs/outputs

The functional performance of the GPIO circuitry was evaluated by establishing interconnected voltage-stimulus channels across the primary digital inputs while simultaneously monitoring relay contact activation. To verify the parametric integrity and operational reliability of the eight general-purpose relay outputs, contacts were characterized for *N* = 100 continuous iterations per general-purpose relay under open-circuit isolation conditions to detect cross-node leakage, and contact resistance measurements were performed to ensure minimal insertion loss during active conduction, additionally, maximum load stress testing was not explicitly performed but it was restricted to operate within the relay’s maximum carry current rating at 1.5 A, per manufacturer specifications) to ensure device reliability.

The performance of the general-purpose inputs was validated by applying high-voltage stimuli ranging from 5 V to 30 V directly to the corresponding pins in the gold-finger edge connector, verifying that the response signals were reliably attenuated to logic-level voltages compatible with the Slave MCUs.

#### 8.1.12 Power-consumption analysis

The system’s power consumption profile was empirically evaluated across three representative operational scenarios: Idle mode, ICT, and FCT. Primary system power is supplied via a ± 15 V dual-rail input delivered through dedicated pins on the modular PCB assembly gold-finger edge connector to the power management module, which drives the high-efficiency LDO regulators to the derived ±5 V and 3.3 V auxiliary rails from this primary supply. These secondary rails power the Main MCU, two Slave MCUs, four relay-based switching matrices (V+, M+, M−, and G− buses), communication interfaces, Digital Multimeter (DMM) sub-circuitry, programmable power supply module, signal-conditioning networks, opto-isolated inputs, general-purpose I/O channels, and additional peripheral onboard electronics. Due to the physical integration and shared power planes of the modular PCB assembly, individual module-level current sensing was not feasible; consequently, power consumption was evaluated across three distinct operational scenarios—Idle mode, ICT, and FCT—by measuring total current draw directly at the primary supply inputs, Table 14 outlines some typical testing methodologies utilized, the observed current consumption varies inherently based on the specific device under test (DUT) and its nominal design parameters.

**Table 14:** Power-consumption analysis for typical tests.

Mode	DUT	Onboard modules used	Current consumption	Power
Idle	no required	Relay-based switching matrices, programmable pull-down module, programmable power supply module, and the DMM (voltmeter module)	170 mA	2.5 W
ICT	Resistance	Relay-based switching matrices, programmable pull-down module, programmable power supply module, comparator module and the DMM (voltmeter module)	295 mA to 340 mA	5 W
	Capacitance	Relay-based switching matrices, programmable pull-down module, programmable power supply module, comparator module and the DMM (voltmeter module)	around 300 mA	4.5 W
	Short- and open-circuit detection	Relay-based switching matrices and the DMM (voltmeter module)	down to 400 mA	6 W
FCT	Voltmeter	Relay-based switching matrices and the DMM (voltmeter module and ammeter module)	down to 210 mA	3.1 W
	Ammeter	Relay-based switching matrices, programmable pull-down module, programmable power supply module and the DMM (voltmeter module)	down to 400 mA	6 W

### 8.2 Automated test equipment implementation

The Electrical Testing Board is integrated into a multi-site Automated Test Equipment framework engineered to execute parallel end-of-line (EOL) functional evaluations across eight concurrent modular PCB assemblies. Within this architecture, each board assembly interfaces with its designated DUT via a dedicated 88-pin gold-finger edge connector, routing signals directly to localized test nodes (pogo pins) embedded on a custom receiver fixture plate. To achieve high-throughput parallel execution, the mechanical fixture is precisely indexed to a multi-cavity DUT grid pallet, where components are seated into isolated test nests. The sequence of the diagnostic routines executed concurrently by each board assembly includes:

1. Initial Provisioning and Bus Telemetry: Execution of the controlled power-up sequence for the DUT, coupled with real-time firmware register extraction and protocol validation via the UART communication interface.
2. Interconnect Continuity and Isolation Profiling: Verification of wire harness alignment, mating integrity, and mechanical connector wiring through dynamic voltage stimuli and open-circuit parametric isolation testing.
3. Parametric Source Metrology: High-resolution evaluation of the internal battery voltage profile under both static no-load (open-circuit) and dynamic transient-load conditions.
4. Quiescent Power Characterization: High-precision metering and profiling of the device’s standby and quiescent current consumption bounds to verify low-power state compliance.
5. Pneumatic Actuation and Node Response Mapping: Synchronization of high-current general-purpose relay outputs to drive external electro-valves (solenoids) for mechanical button activation, paired with concurrent voltage acquisition at designated test nodes to validate switching state transitions.
6. Busbar Presence and Assembly Isolation Verification: Verification of physical battery busbar installation alongside localized short-circuit profiling to detect material or assembly-level structural shunts.
7. Acoustic Transduction and Spectral Verification: Capture of acoustic emissions via the audio-detection module to validate sound pattern integrity and execute frequency-domain or speech-recognition diagnostics.

### 8.3 Conclusions

This paper presents a compact, low-cost, and efficient instrumentation platform designed for concurrent In-Circuit Testing (ICT) and Functional Testing (FCT) of electronic assemblies. The primary contribution of this work centers on a modular printed circuit board assembly (PCBA) architecture that integrates a comprehensive suite of diagnostic capabilities. These include open- and short-circuit isolation profiling, parametric voltmeter, resistance and capacitance metrology, dynamic voltage stimuli generation, analog signal amplification, acoustic transduction validation, and real-time data communication protocols. Fabricated at a nominal component and instrumentation cost of $790.00 USD, the modular PCBA delivers a highly cost-effective alternative to traditional instrumentation within controlled laboratory, prototyping, and low-volume production environments. While industrial-grade commercial ATE (Automated Test Equipment) stations typically demand capital expenditures in the hundreds of thousands of US dollars due to proprietary chassis, licensing, and dedicated instrument cards. Our consolidated architecture offers an open-hardware alternative tailored for academic instruction, circuit prototyping, and small-scale manufacturing, where full-scale testing infrastructure is economically infeasible.

Hardware integration reduces costs by more than 90 % compared to conventional ATE setups. Its flexible design facilitates seamless integration into multi-site frameworks capable of parallel end-of-line execution. This scalable architecture is particularly well-suited to small- and medium-sized enterprises (SMEs) requiring practical quality-assurance infrastructure, though it is not intended to displace high-volume, industrial-grade ICT/FCT systems.

Experimental results validate the system’s functional integrity, throughput, and measurement repeatability. Furthermore, comprehensive power analysis confirms a highly optimized energy profile critical for embedded instrumentation, with a low-power idle state of around 2.5 W during standby and a well-constrained power envelope under operational load during concurrent testing routines. Consequently, this work lowers the barrier to adopting sophisticated electrical testing routines in budget-constrained industrial environments and provides a cost-effective alternative to prohibitively expensive commercial ATE stations. Future research trajectories will focus on integrating TinyML and machine learning principles directly into the firmware layer to optimize test-cycle times and enable predictive fault isolation, and on scaling the physical node architecture to expand signal-routing capacity and test-coverage bounds.

## Ethics statements

The authors have nothing to declare under this heading.

## CRediT authorship contribution statement

**Geu M. Puentes-Conde:** Writing – original draft, Software, Investigation. **Ernesto Sifuentes:** Writing – review & editing, Supervision, Resources, Methodology, Conceptualization. **Javier Molina:** Writing – original draft, Visualization, Supervision. **Francisco Enríquez-Aguilera:** Writing – review & editing, Validation. **Gabriel Bravo:** Writing – review & editing, Validation. **Alejandra Holguín Ávila:** Writing – review & editing, Methodology.

## Declaration of competing interest

The authors declare that they have no known competing financial interests or personal relationships that could have appeared to influence the work reported in this paper.
